# Pulsed antibiotic release into the environment may foster the spread of antimicrobial resistance

**DOI:** 10.1093/femsec/fiaf128

**Published:** 2025-12-19

**Authors:** Matthias Böckmann, Katharina Axtmann, Gabriele Bierbaum, Christiane Zarfl

**Affiliations:** Department of Geosciences, Eberhard Karls University of Tübingen, Schnarrenbergstraße 94-96, 72076 Tübingen, Germany; Institute of Medical Microbiology, Immunology and Parasitology, University Hospital Bonn, Venusberg-Campus 1, 53127 Bonn, Germany; Institute of Medical Microbiology, Immunology and Parasitology, University Hospital Bonn, Venusberg-Campus 1, 53127 Bonn, Germany; Department of Geosciences, Eberhard Karls University of Tübingen, Schnarrenbergstraße 94-96, 72076 Tübingen, Germany

**Keywords:** bacterial competition, half-life, microbial model, minimum selective concentration (MSC), mixture effects, wastewater

## Abstract

The WHO has identified rising antibiotic resistance as a ‘global threat,’ highlighting the urgent need to understand how resistance spreads. A key concept is the minimum selective concentration (MSC)—the threshold at which resistant bacteria gain a competitive advantage. While MSC studies typically use single antibiotics under controlled conditions, real-world environments often contain fluctuating levels and mixtures of antibiotics from sources such as wastewater, complicating the dynamics of resistance spread. This study presents a mathematical model that simulates antibiotic accumulation in aquatic systems to evaluate the resulting influence on resistance selection in microbial communities. It incorporates antibiotic inputs, their photolytic and/or biotic degradation, and microbial competition. Results show that antibiotic accumulation from environmental pulses depends on parameters such as pulse frequency and half-life and may drive the selection of resistant strains. Importantly, combinations of antibiotics significantly alter bacterial competition depending on their interaction type. Synergistic combinations can potentially intensify selection for resistance even when individual antibiotic concentrations are below their respective MSCs. These findings help to understand effects of changing concentrations of multiple antibiotics and to plan mitigation strategies.

## Teaser

Combining experimental data with a mathematical model on microbial dynamics under antibiotic pressure identifies conditions that favour the dominance of resistant over susceptible bacterial strains.

## Introduction

The World Health Organization has classified antimicrobial resistance (AMR) as one of the top 10 global public health threats facing humanity, with recent estimates indicating that, in 2019, bacterial AMR was directly responsible for 1.27 million global deaths and contributed to 4.95 million deaths (Murray et al. 2022). The accelerating emergence and spread of resistance mechanisms threatens to undermine decades of medical advances, rendering routine medical procedures increasingly risky and common infections progressively more difficult to treat (Murray et al. 2022).

While clinical settings have traditionally been the focus of AMR research, there is growing recognition that environmental reservoirs play a critical role in the development and spread of antibiotic resistance (Kümmerer 2009b, Singh et al. 2022). Aquatic ecosystems, which are regularly disturbed by human activities, may provide favourable conditions for the development and spread of AMR, due to the prolonged exposure to low concentrations of antibiotics and favourable growth conditions for bacteria in general (Chow et al. 2021, de Ilurdoz et al. 2022).

Anthropogenically used antibiotics reach the environment via excretions of humans and animals, inappropriate disposal of unused drugs, and direct environmental contamination by waste material from antibiotic production units (Kümmerer 2009a, Kuppusamy et al. 2018, Thai et al. 2018). Most significantly, wastewater treatment plant (WWTP) effluents represent primary conduits for antibiotic release into surface waters, as antibiotics and antibiotic-resistant organisms in the wastewater are generally not or insufficiently withheld (Michael et al. 2013, de Ilurdoz et al. 2022). Additionally, the use of treated or untreated wastewater for irrigation facilitates the spread of these contaminants into soil and crops, raising concerns for environmental and public health (Pan and Chu 2017, Gudda et al. 2023).

The environmental concentrations of antibiotics, while typically below therapeutic levels, still exert significant selective pressure on microbial communities. Even low concentrations of antibiotics can result in the selection of AMR, with responses varying among taxa with different intrinsic resistances, making the establishment of a safe concentration of any antimicrobial compound in the environment challenging (Gullberg et al. 2011, Jechalke et al. 2014, Xiong et al. 2015, Bengtsson-Palme and Larsson 2016). The minimum selective concentration (MSC) is defined as the threshold concentration above which the resistant strain dominates over the susceptible strain, i.e. the dominating strain accounts for more than half of the population. This occurs when the effect of antibiotics on susceptible strains outweighs the cost of resistance, leading to faster growth of resistant bacterial strains.

Furthermore, environmental systems typically contain mixtures of multiple antibiotics and pharmaceuticals, introducing additional complexity to resistance selection dynamics (Oharisi et al. 2023). Synergistic antibiotic interactions demonstrably reduce individual minimum inhibitory concentration (MIC) values through mutual amplification effects (Michel et al. 2008, Fernández Fuentes et al. 2014). Similar synergistic effects at sub-MSC concentrations could intensify selective pressure despite individual compounds remaining below their respective minimum selective thresholds. Conversely, antagonistic effects may mitigate selection pressure despite higher individual concentrations. These combinatorial effects have profound implications for environmental risk assessment, as traditional single-compound approaches may substantially misrepresent actual selective pressures.

A critical but often overlooked aspect of environmental antibiotic exposure is the temporal dynamics of input patterns. The concentrations of antibiotic residues found in wastewater WWTPs make them potential hotspots for antibiotic-resistant bacteria (Che et al. 2019). Unlike laboratory studies that typically employ constant antibiotic concentrations, real-world environments experience pulsed inputs such as those from certain WWTPs with batch operations, agricultural application cycles and industrial discharge patterns (Coutu et al. 2013, Yan et al. 2022). These intervals typically range from a few hours (WWTPs) to multiple days or weeks (irrigation) (Coutu et al. 2013, Christou et al. 2017). The resulting oscillating concentration profiles may lead to antibiotic accumulation depending on input frequency and the half-life of the antibiotic in the environment.

The complexity of these interacting processes—antibiotic input dynamics, environmental fate, microbial growth kinetics, and competition between resistant and susceptible strains—presents considerable challenges for experimental approaches. To complement laboratory and field experiments, there remains a need for a quantitative model describing the concept of the MSC in more complex settings. Mathematical modelling offers a powerful approach to integrate experimental data obtained under controlled conditions with process-based understanding of environmental systems, enabling exploration of scenarios that would be difficult or impossible to replicate experimentally.

This study therefore aims to develop a comprehensive mathematical framework that bridges the gap between laboratory-derived MSC values and realistic environmental exposure scenarios. Specifically, it seeks to: (i) incorporate antibiotic input dynamics and environmental fate processes alongside microbial growth and competition models; (ii) simulate various input scenarios and antibiotic persistence patterns to characterize accumulation behaviour and quantify its impact on microbial resistance dynamics; and (iii) analyse the combined effects of antibiotic mixtures on competition of resistant and susceptible bacterial strains. The competition dynamics under two-antibiotic conditions were validated through laboratory experiments. By integrating these elements within a unified modelling framework, a quantitative tool for assessing resistance risks under environmentally relevant conditions is provided.

## Methods

### General procedure

This study translates the conceptual understanding of microbial competition, antibiotic effects on microbial growth and the spread of AMR into a mathematical model. The model is based on a system of ordinary differential equations and captures the biological and chemical processes driving the dynamics of bacteria and antibiotics. Parameterization and model evaluation were conducted using experimental data as well as insights and data from published literature. The analysis revealed a distinct pattern of antibiotic accumulation under regular pulses, enabling the derivation of an analytical solution.

### Conceptual model

Microbial growth and antibiotic effects were simulated using a system of ordinary differential equations. Two bacterial strains, susceptible (S) and resistant (R), were modelled, competing for a continuously supplied nutrient source. Growth followed Monod kinetics, with nutrient concentration as the primary limiting factor. Growth rates were reduced by bacteriostatic antibiotic effects, modelled using a Hill-type inhibition function incorporating MIC values. A fitness cost was applied to resistant strains, reducing their growth efficiency relative to susceptible bacteria. Temperature and pH influences were considered using empirical growth restriction functions but were excluded from simulations since a potential effect on competitive dynamics could not be identified based on the available experimental data.

Horizontal gene transfer (HGT) was represented in the model structure but excluded from simulations, because resistance in the studied population arises from chromosomal mutation leading to upregulation of an intrinsic efflux pump. Transfer of these mutations was not observed in controls of laboratory experiments (Schuster et al. 2022).

Natural mortality (grazing and other environmental factors) was implemented as a first-order rate. Bactericidal effects were not considered, since sub-MIC concentrations do not result in net killing (Regoes et al. 2004). Nutrient depletion was driven by bacterial uptake proportional to growth, with a constant nutrient inflow assumed. In cases with two antibiotics, combined effects were calculated by summing individual impacts with an additional synergy term.

A selection factor (SF) was introduced to extend the concept of the MSC to cases with two antibiotics and evaluate conditions that favour resistant over susceptible strains. Values of SF ≥ 1 indicated selective advantage for resistance.

Antibiotic pulsing was described analytically by modelling accumulation and degradation dynamics. This led to an oscillatory steady state, from which peak, minimum, and average antibiotic concentrations were derived based on concentration increase, pulse interval, degradation rate, and half-life.

### Model equations

Bacterial growth rates were based on Volkova et al. (2012):


(1) \begin{eqnarray*}
&&{r}_S = S \cdot {\mu }_{max} \cdot \frac{F}{{{K}_m + F}} \cdot {E}_S \cdot {r}_T \cdot {r}_{pH} - \beta \cdot S \cdot R,
\end{eqnarray*}

(2) \begin{eqnarray*}
&&{r}_R = R \cdot {\mu }_{max} \cdot \frac{F}{{{K}_m + F}} \cdot \left( {1 - \alpha } \right) \cdot {E}_R \cdot {r}_T \cdot {r}_{pH} + \beta \cdot S \cdot R,
\end{eqnarray*}

where ${r}_S$ and ${r}_R$  $[ {M{T}^{ - 1}{L}^{ - 3}} ]$ are the specific growth rates of susceptible and resistant bacteria, *S* and *R*  $[ {M{L}^{ - 3}} ]$ are the amount of susceptible and resistant bacteria, ${\mu }_{max}\ [ {{T}^{ - 1}} ]$ is the maximum specific growth rate, $F\ [ {M{L}^{ - 3}} ]$ is the amount of nutrients available, ${K}_m\ [ {M{L}^{ - 3}} ]$ is the Monod-coefficient, ${E}_s$ and ${E}_R\ [ - ]$ are the antibiotic interaction terms for susceptible and resistant bacteria, ${r}_T$ and ${r}_{pH}\ [ - ]$ are the temperature and pH restrictions, $\alpha \ [ - ]$ is the fitness cost, and $\beta \ [ - ]$ is the HGT rate constant (which was set to zero for the dataset used here).

The growth of resistant and susceptible bacteria was defined as the growth rate minus a natural mortality rate, as previously described by experimental studies (Servais et al. 1985, Menon et al. 2003, Gao et al. 2024):


(3) \begin{eqnarray*}
&&\frac{{dS}}{{dt}} = {r}_S - m \cdot S,
\end{eqnarray*}

(4) \begin{eqnarray*}
&&\frac{{dR}}{{dt}} = {r}_R - m \cdot R,
\end{eqnarray*}

where $m\ [ {{T}^{ - 1}} ]$ is the mortality rate constant.

The change of nutrients *F* was defined as


(5) \begin{eqnarray*}
\frac{{dF}}{{dt}} = I - \gamma \left( {{r}_S + {r}_R} \right),
\end{eqnarray*}

where $I\ [ {M{T}^{ - 1}{L}^{ - 3}} ]$ is the constant input of nutrients and $\gamma \ [ - ]$ is the nutrient consumption rate constant.

The antibiotics interaction terms ${E}_i\ [ - ]$ were defined as


(6) \begin{eqnarray*}
{E}_i = 1 - \frac{{{E}_{max}}}{{{{\left( {\frac{{MI{C}_i}}{C}} \right)}}^H + 1}};i = S,R,
\end{eqnarray*}

where $MI{C}_i\ [ {M{L}^{ - 3}} ]$ is the respective MIC for resistant and susceptible bacteria, $C\ [ {M{L}^{ - 3}} ]$ is the antibiotic concentration, and ${E}_{max}\ [ - ]$ and the Hill coefficient $H\ [ - ]$ are shape parameters.

The MSC is defined as the antibiotic concentration where ${r}_S = {r}_R$. For any value of the Hill-coefficient *H*, this leads to


(7) \begin{eqnarray*}
S \cdot \left( {1 - \frac{{{E}_{max}}}{{{{\left( {\frac{{MI{C}_S}}{C}} \right)}}^H + 1}}} \right) = R \cdot \left( {1 - \alpha } \right) \cdot \left( {1 - \frac{{{E}_{max}}}{{{{\left( {\frac{{MI{C}_R}}{C}} \right)}}^H + 1}}} \right)
\end{eqnarray*}

which can only be solved numerically. For *H*=2, the MSC can be analytically defined as


(8) \begin{eqnarray*}
MSC = \sqrt {\frac{{\left( {S - R\left( {1 - \alpha } \right)} \right) \cdot MIC_S^2 \cdot MIC_R^2}}{{{E}_{max}\left( {S \cdot MIC_R^2 - R\left( {1 - \alpha } \right)MIC_S^2} \right) - \left( {S - R\left( {1 - \alpha } \right)} \right)\left( {MIC_S^2 + MIC_R^2} \right)}}}.
\end{eqnarray*}

Temperature and pH growth restrictions were based on Rosso et al. (1995):


(9) \begin{eqnarray*}
{r}_{pH} = \frac{{\left( {pH - p{H}_{min}} \right)\left( {pH - p{H}_{max}} \right)}}{{\left( {pH - p{H}_{min}} \right)\left( {pH - p{H}_{max}} \right) - {{\left( {pH - p{H}_{opt}} \right)}}^2}},
\end{eqnarray*}

where $pH$ is the pH-value during the experiment, $p{H}_{opt}$ is the optimal pH-value, and $p{H}_{min}$ and $p{H}_{max}$ are the lower and upper growth limit for bacterial growth:


(10) \begin{eqnarray*}
{r}_T = \frac{{\left( {T - {T}_{max}} \right){{\left( {T - {T}_{min}} \right)}}^2}}{{({T}_{opt} - {T}_{min})\left[ {({T}_{opt} - {T}_{min}} \right)\ \left( {T - {T}_{opt}} \right) - \left( {{T}_{opt} - {T}_{max}} \right)\ \left( {{T}_{opt} + {T}_{min} - 2T} \right)]}},
\end{eqnarray*}

where *T* is the temperature during the experiment, ${T}_{opt}$ is the optimal temperature, and ${T}_{min}$ and ${T}_{max}$ are the lower and upper growth limit for bacterial growth. All temperatures were expressed in $^\circ C$. The pH and temperature growth factors ${r}_{pH}$ and ${r}_T$ were set to one as a result of the sensitivity analysis (see below).

### Two antibiotics

To account for cases with two antibiotics, the antibiotic interaction terms ${E}_S$ and ${E}_R$ were adjusted loosely based on Nashebi et al. (2024). The effects of antibiotics were summated, and an additional term was added that accounts for synergy:


(11) \begin{eqnarray*}
&&{X}_S = \left( {{\theta }_{11}{C}_1 + {\theta }_{12}{C}_2 + \lambda {\theta }_{11}{\theta }_{12}{C}_1{C}_2} \right),
\end{eqnarray*}

(12) \begin{eqnarray*}
&&{X}_R = \left( {{\theta }_{21}{C}_1 + {\theta }_{22}{C}_2 + \lambda {\theta }_{21}{\theta }_{22}{C}_1{C}_2} \right),
\end{eqnarray*}

(13) \begin{eqnarray*}
&&{\theta }_{11} = \frac{{E_{S1}^{max}}}{{MI{C}_{S1}}};{\theta }_{12} = \frac{{E_{S2}^{max}}}{{MI{C}_{S2}}};{\theta }_{21} = \frac{{E_{R1}^{max}}}{{MI{C}_{R1}}};{\theta }_{22} = \frac{{E_{R2}^{max}}}{{MI{C}_{R2}}},
\end{eqnarray*}

where $E_{S1}^{max}$, $E_{S2}^{max}$, $E_{R1}^{max}$, and $E_{R2}^{max}[ - ]$ are the maximum inhibition rates for antibiotics 1 and 2 towards susceptible (S) and resistant (R) bacteria, $MI{C}_{S1}$, $MI{C}_{S2}$, $MI{C}_{R1}$, and $MI{C}_{R2}[ {\frac{{\mu g}}{l}} ]$ are the MICs of antibiotics 1 and 2 towards susceptible (S) and resistant (R) bacteria, ${X}_S$ and ${X}_R[ - ]$ are the combined effects of antibiotics on the respective bacterial strain, and $\lambda [ - ]$ is the synergy term (positive for synergistic combinations, negative for independent and antagonistic combinations and zero or close to zero for additive combinations). Note that the model was developed for subinhibitory antibiotic concentrations and is not viable for concentrations above the MICs. The effect on bacterial growth was then defined as


(14) \begin{eqnarray*}
{E}_i = \frac{{{E}_{max}}}{{X_i^H + 1}};i = S,R.
\end{eqnarray*}

All other equations were identical to the case with only one antibiotic. Since the concept of the MSC is not viable for combinations of antibiotics—where no single concentration threshold separates dominance of resistant from dominance of susceptible bacteria—the selective outcome depends on the combined concentrations and potential synergy of the antibiotics. Therefore, an SF [−] was defined as


(15) \begin{eqnarray*}
SF = \left( {1 - \alpha } \right) \cdot \frac{{\left( {{X}_S + 1} \right)}}{{\left( {{X}_R + 1} \right)}}.
\end{eqnarray*}

A SF bigger than or equal to one indicates antibiotic concentrations where resistant strains are expected to dominate susceptible strains.

## Generalization of pulses

Regular inputs of antibiotics lead to constant oscillation due to degradation between pulses. Over time, this leads to an ‘oscillating equilibrium’, a dynamic steady-state where antibiotic concentrations fluctuate predictably between defined bounds. If this ‘oscillating equilibrium’ is reached, the concentration increase following a pulse must be equal to the antibiotic degradation between pulses:


(16) \begin{eqnarray*}
{\mathrm{\Delta }}C = {C}_{max} - {C}_{max} \cdot {e}^{ - k \cdot {t}_P}
\end{eqnarray*}

with:


(17) \begin{eqnarray*}
k = \frac{{\ln \left( 2 \right)}}{{{t}_{\frac{1}{2}}}},
\end{eqnarray*}

where ${\mathrm{\Delta }}C\ [ {M{L}^{ - 3}} ]$ is the concentration increase due to a pulse, ${C}_{max}\ [ {M{L}^{ - 3}} ]$ is the upper limit of the oscillation caused by pulses, $k\ [ {{T}^{ - 1}} ]$ is the degradation rate constant of the respective antibiotic, ${t}_P\ [ T ]$ is the time between pulses, and ${t}_{\frac{1}{2}}\ [ T ]$ is the half-life of the respective antibiotic. Solved for ${C}_{max}$, this led to


(18) \begin{eqnarray*}
{C}_{max} = {\mathrm{\Delta }}C \cdot \frac{1}{{1 - {2}^{ - n}}}
\end{eqnarray*}

with


(19) \begin{eqnarray*}
n = \frac{{{{{t}}}_{\mathrm{P}}}}{{{t}_{\frac{1}{2}}}}.
\end{eqnarray*}

The average concentration $\bar{C}\ $was then defined as


(20) \begin{eqnarray*}
\bar{C} = \frac{{\mathop \smallint \nolimits_0^{{{\mathrm{t}}}_{\mathrm{P}}} {C}_{max} \cdot {e}^{ - k \cdot t}\ dt}}{{{{{t}}}_{\mathrm{P}} - 0}}
\end{eqnarray*}

which led to


(21) \begin{eqnarray*}
\bar{C} = {\mathrm{\Delta }}C \cdot {t}_{\frac{1}{2}} \cdot \frac{1}{{\ln \left( 2 \right) \cdot {t}_P}}.
\end{eqnarray*}

${C}_{min}\ [ {M{L}^{ - 3}} ]$ is the lower limit of the oscillation and equals the maximum concentration minus ${\mathrm{\Delta }}C$:


(22) \begin{eqnarray*}
{C}_{min} = {C}_{max} - {\mathrm{\Delta }}C = {\mathrm{\ \Delta }}C \cdot \left( {\frac{1}{{1 - {2}^{ - n}}} - 1} \right).
\end{eqnarray*}

A more detailed derivation including intermediate steps can be found in the Supplementary Data.

### Generalization across antibiotics based on MSC and half-life

The two processes ‘balancing’ the effective antibiotic concentration, i.e. the concentration above the MSC, are the antibiotic concentration increase by the input pulse and the degradation between two pulses. To generalize the potential risk of resistant bacteria outcompeting the susceptible strain across different half-lives and antibiotics, probability distributions for antibiotic concentrations (${{\bf \Delta }}{\boldsymbol{C}}$, concentration increase, and ${\boldsymbol{\bar{C}}}$, average concentration) and half-lives ${{\boldsymbol{t}}}_{1/2}$ were constructed from available literature (see Table 1). These distributions were visualized in a two-dimensional parameter space, combining the input extent with MSC and the input dynamics with degradation. Parameter combinations for which resulting average concentrations exceeded MSC thresholds were identified as high-risk conditions for resistance development. Two degradation pathways were distinguished that focus on different environmental conditions and thus specific degradation processes, i.e. photolytic degradation and biodegradation. For the photolytic degradation pathway, a system exposed to direct sunlight and thus focusing on photodegradation containing clean water with minimal bacterial presence and chemical compounds was simulated. Half-lives have been compiled from published studies that investigated these specific conditions (see Table 1). For the biotic degradation pathway, a system without sunlight exposure but with high concentrations of bacteria and chemical compounds typically found in wastewater was simulated. Half-lives have therefore been derived from studies of comparable systems under similar conditions. All other parameters remained identical to the photolytic degradation pathway.

**Table 1.** Mean values derived from literature and deviations of parameters describing environmental concentrations, MSC, and degradation half-lives under photolytic conditions and under conditions favourable for biodegradation.

Antibiotic	Concentration [µg/l]	MSC [µg/l]	${t}_{1/2}$ [h] (Photolytic)	${t}_{1/2}$ [h] (Biotic)	Effect
Ciprofloxacin	$3.2 \pm 1.1$	$16.7 \pm 1.1$	$23 \pm 23$	$208 \pm 170$	Bactericidal
Erythromycin	$2.2 \pm 0.7$	$514 \pm 93$	$18.6 \pm 13.3$	$480 \pm 353$	Bacteriostatic
Levofloxacin	$7.1 \pm 2.3$	$43 \pm 17$	$1.6 \pm 1.0$	–	Bactericidal
Metronidazole	$0.7 \pm 0.2$	$16 \pm 3$	$5.1 \pm 0.8$	–	Bactericidal
Norfloxacin	$1.2 \pm 0.4$	$16 \pm 3$	$0.02 \pm 0.005$	$363 \pm 238$	Bactericidal
Ofloxacin	$1.0 \pm 0.3$	$8.0 \pm 1.5$	$34 \pm 23$	$266 \pm 195$	Bactericidal
Sulfamethoxazole	$4.1 \pm 1.4$	$298 \pm 28$	$3.2 \pm 2.7$	$1078 \pm 770$	Bacteriostatic
Trimethoprim	$1.8 \pm 0.6$	$16 \pm 3$	$17 \pm 13$	$526 \pm 458$	Bacteriostatic

The concentration data originate from measurements in wastewater. All parameters refer to antibiotics in aquatic environments. Deviations are based on the 2.5th and 97.5th percentile, respectively. Missing values indicate that no data were found. Detailed citations for shown parameters are listed in the Supplementary Data.

### Nondimensionalization

To enable a direct comparison across antibiotics, concentrations were normalized by their respective MSCs, and pulse intervals ${t}_P$ were normalized by antibiotic half-lives ${t}_{\frac{1}{2}}$. The uncertainties of those parameters were propagated accordingly. This allowed for comparison of resistance risks for different antibiotics on the same scale. Note that this was not a representation of combinations of antibiotics but a comparison of the individual effects of antibiotics.

### Underlying data

For conceptual model simulations, parameters regarding bacterial growth and nutrients were obtained from controlled laboratory experiments. Environmental factors were only considered for bacteria; half-lives of antibiotics were assumed to be constant within the confidence intervals and different degradation pathways.

Additionally, the model was applied to analyse two different cases:

First, simulations were compared with selected antibiotics to generalize the effect of pulse intervals and pulse concentrations on the resulting environmental concentrations in relation to the MSC. Environmental antibiotic concentrations, MSCs, and half-lives were compiled from the scientific literature (see Table 1). The uncertainties of $MSC$ and ${t}_{1/2}$ were characterized using the 2.5th and 97.5th percentiles. In cases where no sufficient data were available, the uncertainty was estimated based on the relative deviation derived from cases where data were available. Pulse intervals ${t}_P$ were set to vary between 24 and 48 h. Pulse concentrations ${\mathrm{\Delta }}C$ were based on the arithmetic mean of literature concentrations observed in wastewater with the same relative deviation as the pulse intervals ${t}_P$ (one third of the mean value).

Second, the model structure considering two antibiotics was applied to experimental data to investigate antibiotic synergy. The details of the experimental procedure are described in the following.

### Experimental procedure and analysis to investigate antibiotic synergy

#### Bacterial strains

*Acinetobacter baylyi* green fluorescent protein (GFP) and *A. baylyi* mCherry 652 were used for all experiments described. Both strains were constructed by Schuster et al. (2022) and are derived from *A. baylyi* BD413. This strain was transformed with a gene cassette encoding the fluorescent proteins GFP or mCherry and a resistance-gene for spectinomycin. *A. baylyi* mCherry 652 was selected as a spontaneous mutant showing decreased susceptibility to various antibiotics, probably due to a deletion of the *adeN* gene leading to an upregulation of the AdeIJK efflux pump (Schuster et al. 2022). *A. baylyi* was chosen as a model organism, since it naturally occurs in wastewater, sewage sludge, and soils, i.e. environments that are directly relevant for the application of the provided model. In addition, the genus *Acinetobacter* is of high clinical importance due to the pathogenic species *A. baumannii*, making *A. baylyi* a suitable model organism for clinical relevance. Efflux pump upregulation provides a simple and ecologically realistic mechanism conveying decreased susceptibility to multiple antibiotics, which is necessary if resistance to combinations of compounds is explored. In addition, many clinical isolates contain such upregulated efflux pumps (Blair et al. 2014).

#### Checkerboard fractional inhibitory concentration for two antibiotics

To determine the fractional inhibitory concentration (FIC) values for combinations of different antibiotics, checkerboard assays were performed for each strain (*A. baylyi* GFP and *A. baylyi* mCherry 652). The determination followed the guidelines for broth microdilution method of the Clinical and Laboratory Standards Institute in 96-well polystyrene round-bottomed microplates (Greiner Bio-One International GmbH, Kremsmünster, Austria) in Mueller–Hinton broth (Oxoid Limited, Basingstoke, United Kingdom) with a final concentration of 5 × 10^5^ cells per ml. The plates were incubated for 20 h at 30°C and 180 r/m. The FIC values were determined as previously described by Tam et al. (2004). Antibiotic combinations with FIC values ≤0.5 were categorized as ‘synergistic’, combinations with FIC values between 0.5 and 1.0 as ‘additive’, between 1 and 4 as ‘independent’, and combinations with FIC values >4 as ‘antagonistic’. A minimum of three independent measurements were performed for each strain and antibiotic combination. The selection of the antibiotic combination was based on the requirement that the resistant strain (mCherry 652) shows reduced susceptibility to both tested antibiotics, enabling a direct comparison with the susceptible strain (GFP). Among the available options, the antibiotic combination trimethoprim-sulfamethoxazole represented the combination with the lowest FIC-value (0.4), sulfadiazine-sulfamethoxazole resulted in an FIC of ∼1 and chloramphenicol-sulfamethoxazole yielded the highest FIC values (1.5). Hence, these three combinations provided representative cases for synergistic, additive, and independent interactions.

#### Checkerboard MSC determinations

MSC for two antibiotics simultaneously were tested in checkerboard assays. The maximum concentration of each antibiotic corresponded to the fourfold MSC of the respective antibiotic alone, as previously described for *A. baylyi* GFP and *A. baylyi* mCherry 652 by Schuster et al. (2022). All experiments were performed in a total volume of 200 µl in black 96-well f-bottomed polystyrene microplates with sterile transparent plastic lids (Greiner Bio-One International GmbH, Kremsmünster, Austria). An inoculum of both bacterial strains (*A. baylyi* GFP and *A. baylyi* mCherry 652) was added to each well aiming for a final concentration of 2.5 × 10^5^ cells per ml for each strain. The plates were incubated for 20 h at 30°C and 180 r/m. All plates included growth controls for both strains as well as a sterile control.

Antibiotic synergy was tested with sulfamethoxazole in combination with trimethoprim, sulfadiazine, and chloramphenicol. The experimental design incorporated 5, 8, and 5 biological replicates, respectively, with duplicate technical measurements performed for each biological replicate.

For fluorescence measurements, images of well plates were processed as follows: the images were first aligned and segmented into individual well images, which were clipped to exclude background pixels. All pixels with a brightness below a manually chosen threshold were excluded as well. The remaining pixel intensities for each well were averaged separately for the red and green bands. The resulting averages were normalized by dividing all values by the highest observed intensity in the respective band.

The normalized red-to-green ratio was computed for each well to estimate the ratio of resistant to susceptible (R/S ratio) bacterial populations. This ratio was log-transformed for improved visualization and statistical analysis.

### Sensitivity and uncertainty analysis

A local sensitivity analysis of the parameters used in the ordinary differential equations (bacterial growth and nutrients) was performed for two scenarios by varying parameters by $\pm 10\%$ to test effects on the simulation results. One scenario simulates an antibiotic concentration below the MSC, the other scenario simulates an antibiotic concentration above the MSC.

For the accumulation of antibiotics, the concentration increase ${\mathrm{\Delta }}C$ and the pulse intervals ${t}_P$ were randomly perturbed over time to assess the effect of irregular pulses of varying amounts of antibiotics on the accumulation.

Additionally, a global sensitivity analysis was conducted to evaluate the relative importance of multiple parameters simultaneously across their entire value range. Unlike local sensitivity analysis, which assesses the impact of small perturbations around a nominal value, the global sensitivity analysis accounts for interactions between parameters and their influence on the overall system behaviour. The method for the global sensitivity analysis was based on the active subspace method, which consists of a principal component analysis for derivatives of the objective function in the parameter space (Constantine and Diaz 2017). Details on the implementation can be found in Boeckmann et al. (2025). The objective functions of all sensitivity analyses were the arithmetic mean of susceptible and resistant bacteria, respectively, and the normalized root mean square error (NRMSE) for the case with two antibiotics.

The bootstrapping algorithm was used to determine the uncertainty of the calibration (Efron 1992). Bootstrapping can be viewed as a smoothed variant of cross-variance (Efron and Tibshirani 1997) and is based on generating multiple sub-datasets (bootstrap samples) by resampling the original dataset with replacement. Each of these bootstrap datasets was used to fit the model, producing a unique set of parameter estimates. Collectively, these parameter sets formed a posterior distribution, which served as a basis for quantifying the uncertainty of the estimated parameters (95% confidence interval).

All simulations and analyses were performed with MATLAB code specifically developed for this purpose (The MathWorks, Inc. 2023).

## Results and discussion

### Pulsed antibiotic inputs and bacterial dynamics

**Figure 1.**
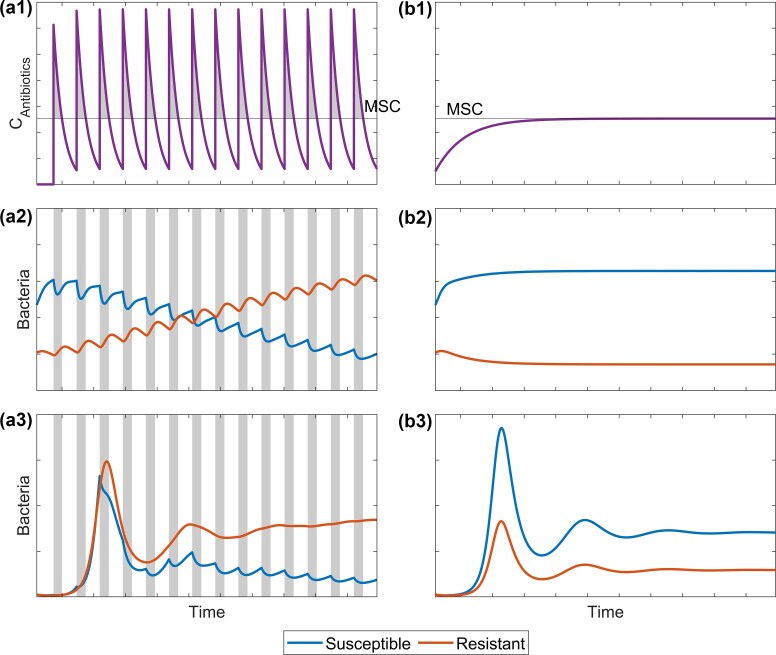
Conceptual simulation of effects of antibiotic input dynamics on antibiotic concentrations in an aquatic system based on equations 16–22 and subsequent dynamics of resistant and susceptible bacteria of the same strain based on equations 1–15. (a) Oscillation and accumulation of antibiotics over time may lead to resistant bacteria outcompeting the susceptible strain. Grey bars indicate periods of antibiotic concentrations exceeding the MSC. (b) Continuous accumulation of antibiotics below the MSC due to continuous input and degradation supports the dominance of the susceptible bacterial strains. (a3) and (b3) same conditions as (a2) and (b2), respectively, with lower starting populations of bacteria.

Antibiotic pulses were simulated by sudden increases of the antibiotic concentration (${\mathrm{\Delta }}C,$ Table 1) followed by a ‘recovery phase’ defined by the pulse interval ${t}_P$, during which the antibiotic concentration is reduced according to its half-life ${t}_{1/2}$. This led to an accumulation in the beginning followed by a periodical oscillation of the antibiotic concentration. In the shown example (Fig. 1), the concentration increase ${\mathrm{\Delta }}C$, the pulse interval ${t}_P$ and the half-life ${t}_{1/2}$ were chosen to simulate an oscillation with the average concentration equal to the MSC. Hence, the oscillation led to concentrations of antibiotics that exceeded the MSC substantially followed by concentrations below the MSC (Fig. 1a). For comparison, a scenario with continuous inputs was simulated with the same average concentration at equilibrium (see Fig. 1b). In scenario (a) (pulsed inputs), the resistant strain was dominating, while the susceptible strain was suppressed, when the antibiotic concentration was above the MSC (grey shaded areas). This effect resulted from reduced bacterial growth rates due to antibiotic exposure and natural mortality, since bactericidal effects were not simulated. In scenario (b) (continuous inputs), the susceptible strain was dominating due to antibiotic concentrations below the MSC during accumulation. The same input pattern was evaluated with different initial condition for the bacterial strains (Figs. a2/b2 vs. a3/b3). When the antibiotic concentration reached the MSC, the susceptible strain maintained dominance due to its already larger population (b). This shows that antibiotic pulses favour resistant strains more than the resulting average concentration might suggest, since the intervals with an antibiotic concentration above the MSC led to a stronger disadvantage for the susceptible strain and is not compensated by the advantage of the susceptible strain during periods with a concentration lower than the MSC. This effect is independent of the initial bacterial population, although the time required to reach equilibrium may vary depending on the initial conditions. The exact magnitude of the effect depends on the amplitude of the concentration oscillations. Under the simulated conditions, this led to a shift in the effective mean MSC by approximately 5%–15%.

This emphasizes the importance of understanding the dynamics of periodic inputs. In general, the continuous release of low concentrations should be preferred, if release into the environment is unavoidable. WWTPs generally discharge effluent relatively continuously, resulting in small short-term fluctuations (Coutu et al. 2013). However, pronounced diurnal peaks in antibiotic concentrations have been observed in raw hospital wastewater, with ciprofloxacin and meropenem exhibiting reproducible morning and evening maxima (Schuster et al. 2022). On longer timescales, substantial seasonal peaks occur in temperate regions, particularly in winter when higher prescription rates lead to higher antibiotic loads (McArdell et al. 2003; H. Zhang et al. 2023). In addition, storm events and combined sewer overflows can also lead to fluctuations of antibiotics (Dong et al. 2019, Furrer et al. 2023). Such temporal oscillations, ranging from hours to months, can periodically raise antibiotic concentrations above the MSC, creating the same patterns as shown in the simulations and thereby promoting the selection of resistant strains even when time-averaged concentrations remain below the MSC.

### Normalized simulation of antibiotic input and degradation dynamics

**Figure 2.**
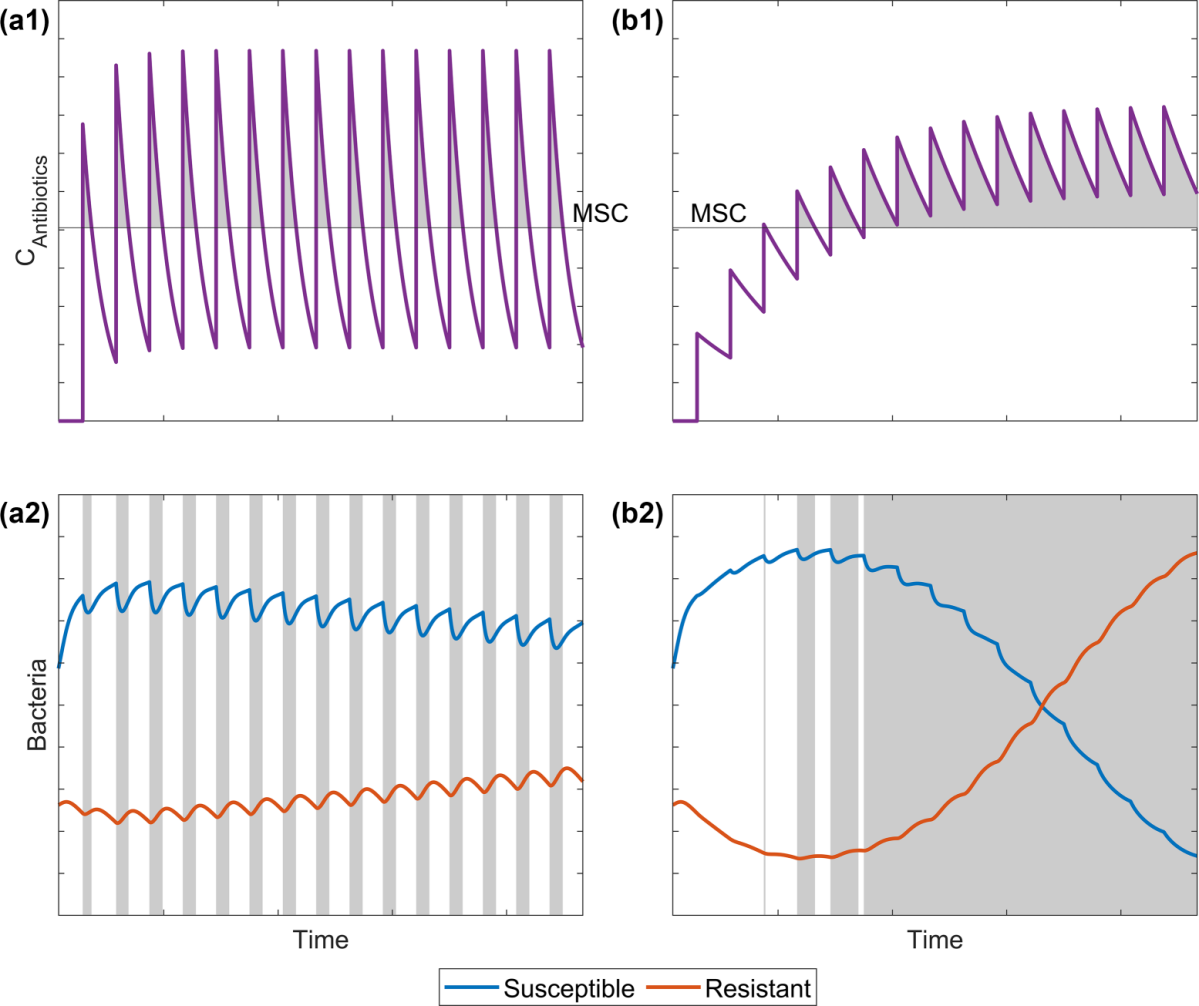
Conceptual simulation of effects of accumulation on antibiotic concentrations in an aquatic system based on equations 16–22 and subsequent dynamics of resistant and susceptible strains based on equations 1–15. Grey areas indicate periods of antibiotic concentrations exceeding the MSC. (a) Oscillating antibiotic concentrations over time with minor accumulation led to minor advantages for the resistant strain which may lead to a dominance of the resistant strain after long exposure times. (b) Oscillating antibiotic concentrations over time with strong accumulation led to a strong increase and subsequent dominance of the resistant strain.

**Figure 3.**
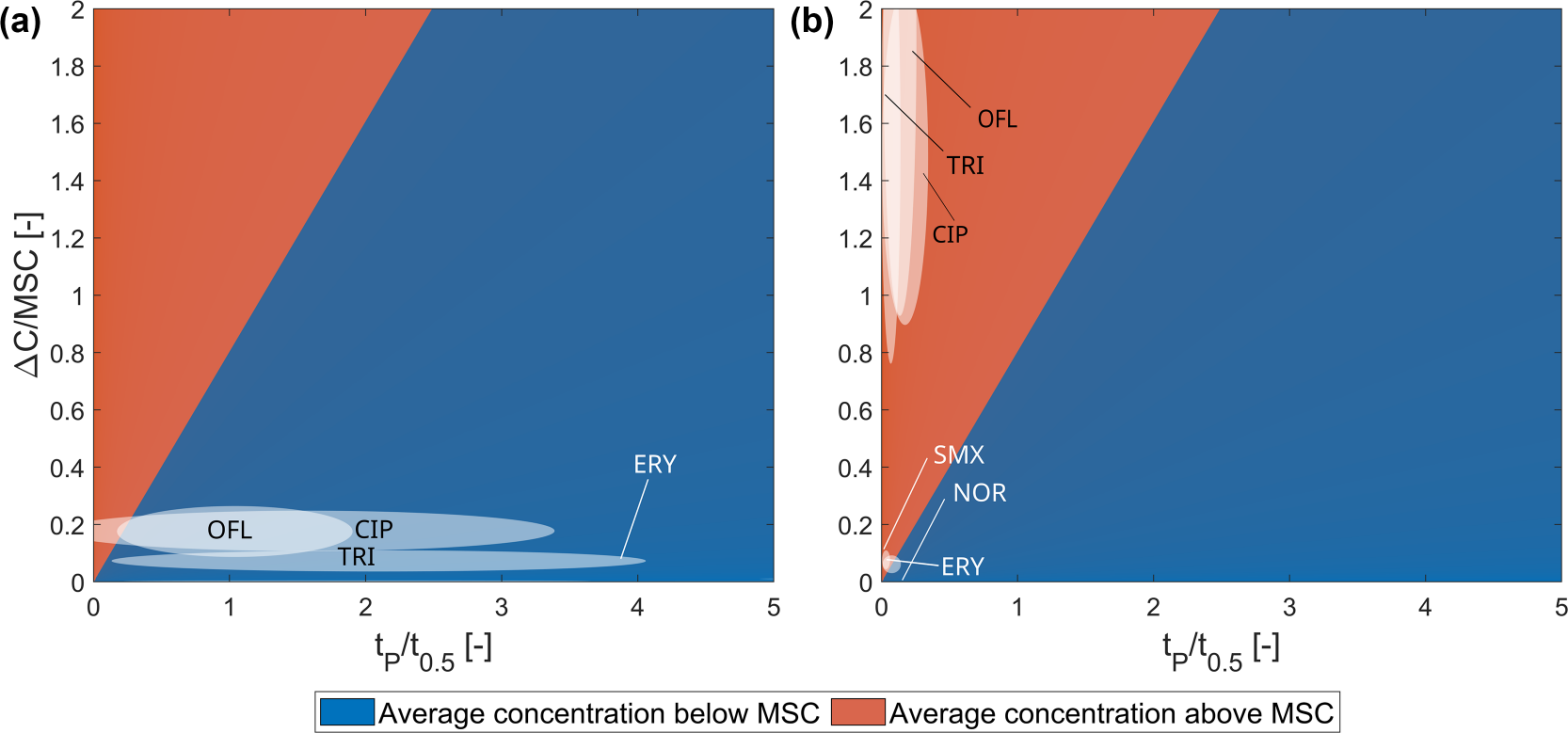
Simulated dependence of the average antibiotic concentration on the input pulse on the concentration increase in relation to the MSC and the pulse interval in relation to the degradation half-life of the respective antibiotic, based on literature data (Table 1) and the antibiotic pulse equations (equations 16–22). Photolytic degradation is shown in the left tile; (a) biotic degradation is shown in the right tile (b). The red area shows parameter combinations where the resulting average concentrations are above the MSC. Blue areas show parameter combinations where the resulting average antibiotic concentrations are below the MSC. Confidence intervals of resulting average concentrations of selected antibiotics based on concentration increase **ΔC**, MSC, pulse times *t*_P_, and half-lives *t*_1/2_. CIP, ciprofloxacin; ERY, erythromycin; MET, metronidazole; OFL, ofloxacin; SMX, sulfamethoxazole; and TRI, trimethoprim.

A conceptual simulation of pulsed inputs showed oscillating antibiotic concentrations with either low accumulation (a) or strong accumulation (b) (Fig. 2). In the first case, the resulting mean concentration equalled the MSC. If represented in the chemical space plot (Fig. 3), this scenario would lie directly on the line separating the red from the blue area. In the second case, the antibiotic concentration is initially low but increases over time due to accumulation, ultimately resulting in a mean concentration substantially above the MSC and dominance of the resistant strain after a short time. This scenario would be located in the red area of the chemical space plot.

The normalization of input extent and dynamics in relation to the antibiotic half-lives and MSC was illustrated in a chemical space plot (Fig. 3). The resulting antibiotic concentration was calculated based on equation 21 incorporating the accumulation parameters: MSC, half-life ${t}_{1/2}$, concentration increase $\Delta C$, and pulse interval ${t}_P$ specific to each antibiotic (Table 1). Parameter combinations for which the resulting average concentration exceeded the MSC are marked as red. All other parameter combinations are shown in blue. The line separating the red and blue area indicates combinations of accumulation parameters that result in average antibiotic concentrations equal to the MSC. Note that this line is not determined by antibiotic properties, but from the relationship between the concentration increase $\Delta C$ and resulting average concentration $\bar{C}$, as specified in equation 21. According to this equation, the slope of this line equals $\ln ( 2 )$.

Photolytic degradation. Under photolytic degradation conditions, the probability distributions of the accumulation parameters of most investigated antibiotics in the chemical space plot lay predominantly within the blue area, indicating that their average concentrations are expected to remain below the MSC (Fig. 3a). An exception is ciprofloxacin (CIP), whose distribution partially overlaps with the red area. Additionally, trimethoprim (TRI) and ofloxacin (OFL) exhibit distributions that are near this boundary.

These findings suggest that, compared to the other antibiotics examined, ciprofloxacin, ofloxacin, and trimethoprim may pose a relatively higher risk of exceeding the MSC under the assumed concentration increase $\Delta C$ and pulse interval ${t}_P$, and therefore of promoting the dominance of the resistant bacteria strain. In contrast, the remaining antibiotics undergo a fast degradation, i.e. their half-lives for extensive photolytic degradation are short in relation to the pulse interval. Consequently, these parameter distributions are located far to the right on the *x*-axis and fall outside the red area. They are therefore not shown in Fig. 2.

Based on the available data, it appears unlikely that the investigated antibiotics will exceed MSC thresholds under environmental conditions that are favourable for photodegradation. Nevertheless, site-specific factors (e.g. sunlight intensity, water quality, and local antibiotic usage patterns) could shift an antibiotic’s probability distribution closer to the red area. For example, photolytic degradation can be both, enhanced and reduced, when the antibiotic residues are sorbed to suspended particles present (Fatta-Kassinos et al. 2011), or other organic and inorganic chemicals present in wastewater or irrigated soils. Felis et al. (2022) reported a removal of 52.4% of the before examined antibiotics and 83.3% removal of antibiotic resistance genes in treated wastewater after irradiation with solar light. Especially in low-income countries in warmer climates, waste stabilization ponds can be used using the natural sunlight to remove antibiotic residues at low costs (V. M. Starling et al. 2021, Carpanez et al. 2025). The results presented here highlight the important influence of photolytic degradation of antibiotic residues on selective concentrations (Boreen et al. 2003, Homem and Santos 2011).

Biodegradation. Under environmental conditions that favour biodegradation, the probability distributions of ciprofloxacin (CIP), erythromycin (ERY), ofloxacin (OLF), sulfamethoxazole (SMX), and trimethoprim (TRI) were predominantly or entirely located in the red area (i.e. average concentration above MSC) (Fig. 3b). These differences arose solely from the adjusted half-lives of the respective antibiotics, as half-life was the only parameter altered between degradation pathways. Since degradation of fluoroquinolones (ciprofloxacin, ofloxacin, and norfloxacin), as well as trimethoprim, sulfamethoxazole, and erythromycin, relies heavily on photolytic degradation pathways (Table 1), the relatively low biotic degradation rates explain the substances’ presence in the red area when photolysis does not play a substantial role. Despite having a biotic half-life comparable to other fluoroquinolones, norfloxacin did not appear in the red area due to its low environmental concentrations relative to its MSC (Table 1). Levofloxacin was not included in the analysis, but its location in the space plot is expected to resemble that of the other fluoroquinolones.

The results of these calculations can help planning mitigation strategies by quantifying how modifications in antibiotic release influences overall risk. For instance, increasing the pulse interval ${t}_P$ would shift the entire distribution to the right (towards the blue area). Since the relationship between the pulse time and the resulting mean concentration $\bar{C}$ is linear (see equation 21), doubling the pulse time would shift all antibiotics to the right by a factor of two. However, this risk-reduction effect is effectively neutralized if the pulse concentration is simultaneously doubled while maintaining the total antibiotic load. The probability distribution is shifted along the MSC-line, meaning the same ratio remains within the red and blue area. Thus, redistributing the temporal pattern of release alone cannot fundamentally alter the resulting mean concentration.

Although a previous study by Vogel et al. (2023) indicated that high pulse concentrations might enhance pollutant degradation, this was attributed to solubility effects relevant to polycyclic aromatic hydrocarbons, which does not apply to the antibiotic concentrations investigated here.

Simulations suggest that higher pulses increase the risk of resistance (Fig. 1), as the polynomial concentration term in the antibiotic interaction function (see equation 6), leads to a substantial disadvantage on susceptible bacteria at high concentrations. It should be noted that the concentrations considered ‘high’ in this environmental context remain far below the MIC and fall within the lower end of the selective window (Gullberg et al. 2011), so these pulses amplify the selective pressure that favours resistant strains. Depending on intrinsic resistances, also the response of different bacterial taxa to antibiotic concentrations in the environment differs greatly and antibiotic pulses might also favour growth of intrinsically resistant taxa and lead to shifts in the bacterial community composition (Reichel et al. 2013). Hence, if the total antibiotic load cannot be reduced, the most promising strategy is to release it as continuously as possible, i.e. to increase the application frequency while reducing peak concentrations, even if this approach shortens the potential recovery periods between pulses.

A study by Morsky and Vural (2022) found that pulsed antibiotic inputs could prevent resistant strains from dominating the total population. However, the scenario shown in that study involved a substantially lower total antibiotic load than the continuous application scenario. Hence, this does not contradict the advantage of a continuous application of a given amount of antibiotics.

### Mixtures of two antibiotics

**Figure 4.**
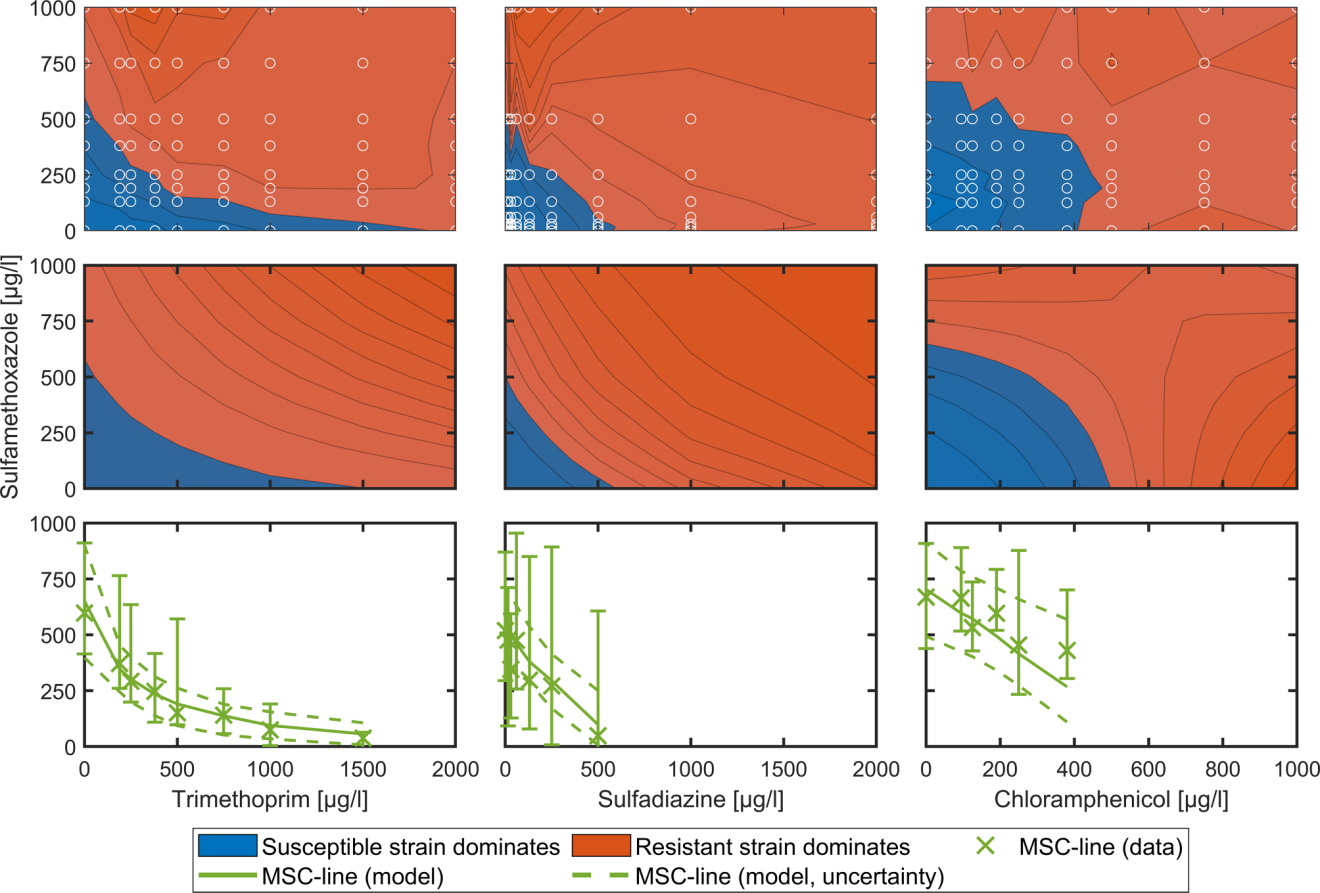
Overview of combinations of trimethoprim, sulfadiazine, and chloramphenicol with sulfamethoxazole with respective FIC values of 0.4, 1, and 1.5. The first row shows results from microbial growth experiments, displayed as log₁₀ *R*/*S* ratios. Circles mark the positions of the experimental data points, which represent observed values. Areas between the circles are interpolated. The second row shows simulated log_10_  *R*/*S* ratios based on the biological growth model (equations 1–15). The third row compares the resulting MSC lines from data and simulations, including 95% confidence intervals for both. The FIC values of combinations increase from left to right.

Experiments were conducted using sulfamethoxazole in combination with trimethoprim, sulfadiazine, and chloramphenicol to characterize their pharmacodynamic interactions and validate the model (Fig. 4). The model was calibrated exclusively to the MSC-trajectory [log10 (*R*/*S*) = 0, transition from blue to red]. Hence, the model results are only valid around this trajectory.

Antibiotic combinations showed increasing FIC-values from left to right, revealing distinct interaction patterns aligned with theoretical expectations. The trimethoprim-sulfamethoxazole combination showed characteristic convex patterns consistent with low FIC values (<0.5) confirming the known synergistic interaction of the two antibiotics (Knothe 1978), while the chloramphenicol-sulfamethoxazole combination showed concave patterns reflecting high FIC values and independent effects. Overall, the results are consistent with statistical modelling results regarding the synergy of the MIC (Michel et al. 2008). In comparison, the trimethoprim-sulfamethoxazole combination exhibited substantially greater resistance selection pressure than either sulfamethoxazole-sulfadiazine or sulfamethoxazole-chloramphenicol pairings, with selection pressure substantially exceeding the additive effects of individual compounds. This raises concerns, as the observed synergistic selection represents a combination of only two antibiotics, whereas environmental cases frequently contain complex mixtures of multiple antibiotics acting simultaneously on microbial communities (de Ilurdoz et al. 2022). Still, antibiotic responses may vary between different taxa and are influenced by physicochemical properties of the environment (e.g. sorption in soil) as well as other biotic and abiotic environmental factors (Jechalke et al. 2014). Moreover, synergistic combinations of antibiotics are commonly administered together and therefore, both substances are excreted together into the wastewater system. While synergistic antibiotics greatly promote existing resistance, the scientific literature presents contradictory findings regarding their influence on spontaneous mutations to resistance (Michel et al. 2008, Lozano-Huntelman et al. 2025). Additionally, these findings raise questions regarding antibiotic concentrations, that are considered to be non-hazardous (e.g. PNECs) which are traditionally calculated for single antibiotics and not for combinations of two or more substances (Bengtsson-Palme and Larsson 2016, Booth et al. 2020). Risk assessment for antibiotic combinations found commonly together in the environment are needed, as also additive combinations—like the here tested sulfamethoxazole-sulfadiazine—lower the single concentrations needed to select for resistance.

Notably, the chloramphenicol combination demonstrated rapid convergence toward the *x*-axis due to its pronounced concave profile, resulting in early termination of the MSC trajectory as subsequent observations showed complete absence of susceptible bacterial dominance.

Model quality assessment showed low NRMSE values of 0.09, 0.15, and 0.10 for the respective combinations, calculated from differences between modelled and observed MSC trajectories. This robust agreement between theoretical predictions and experimental observations suggests that comprehensive parameterization—incorporating MIC, fitness cost coefficient (α), and maximum antibiotic inhibition rates—could enable accurate prediction of MSC-trajectories and the SF. Such predictive capability could substantially reduce experimental complexity while providing quantitative risk assessment tools for environmental monitoring, particularly if model parameters can be generalized across bacterial strains and different antibiotics.

### Effects of environmental factors

All relevant environmental factors (pH, temperature, and nutrient availability) and bacterial growth parameters (maximum growth rate, Monod constant) were systematically varied to assess their impact on competition between susceptible and resistant bacterial strains. Except for the MIC, the fitness cost $\alpha $ and the maximum antibiotic inhibition rates, all parameters were set to identical values for both strains. The results indicate that, aside from nutrient availability, none of the tested parameters influence the *R*/*S* ratio. Specifically, elevated nutrient levels shifted the *R*/*S* ratio slightly towards resistance at antibiotic concentrations above the MSC and towards susceptibility below the MSC. This is most likely caused by the unique role of nutrient availability in modulating competition between both strains. Other factors (except MICs and fitness cost $\alpha $) affect bacterial growth as well, but not the competition or the *R*/*S* ratio.

Since nutrient availability in natural systems is generally lower than in laboratory settings, this should be considered when conclusions about natural systems are drawn from laboratory experiments. Nonetheless, MSC values determined under controlled laboratory conditions appear to remain valid as an indicator of resistance risk.

Since pH and temperature did not affect the *R*/*S* ratio, effects from these parameters were excluded from other simulations.

Environmental factors were only considered for their direct effect on bacteria. Environmental effects on antibiotics were not considered. However, environmental fate of antibiotics is fundamentally governed by sorption to soil or other solid matrices, which affects their mobility, persistence, and bioavailability (Jechalke et al. 2014, Cycoń et al. 2019). While biotic effects of antibiotics (growth limitation, synergy) are included in the model, these chemical effects are restricted to degradation and accumulation. Strong chemical differences are shown between highly sorbing antibiotics, such as trimethoprim and the fluoroquinolones, which show strong affinity for soil organic and mineral surfaces leading to accumulation and extended persistence in the solid phase (Y.-L. Zhang et al. 2014), and weakly sorbing compounds including sulfamethoxazole and sulfadiazine, which display only limited interaction with soil components (e.g. humic substances, organic particulates, and clay minerals) (Y.-L. Zhang et al. 2014, Hu et al. 2019). While elevated sorption generally extends residence time in soils, its influence on degradation remains inconsistent across studies (Famisan and Brusseau 2003, Wu et al. 2011, Buerge et al. 2020), potentially attributable to varying sorption mechanisms or inadequate differentiation between dissipation and true degradation or mineralization processes (Ukalska-Jaruga et al. 2023, Boeckmann et al. 2025). This complexity is further increased by other environmental factors, e.g. pH-value and competitive sorption (Park and Huwe 2016, Septian et al. 2019). In addition, half-lives differ substantially between photolytic and biotic conditions (Table 1 and Fig. 3), with many antibiotics showing rapid removal under sunlight but persistence when photolysis is absent. Under conditions where sorption to soils or particles is relevant, the contribution of photolytic degradation is further reduced, making biotic degradation the dominant pathway (Ozaki et al. 2011). The differential sorption and degradation characteristics translate into varying selection pressure profiles, where highly sorbed antibiotics may exert reduced short-term but potentially persistent long-term selection pressure, while weakly sorbed antibiotics impose higher immediate pressure over shorter temporal scales. This is especially relevant for combinations of antibiotics that exhibit markedly different sorption properties (i.e. trimethoprim and sulfamethoxazole).

### Sensitivity analysis

The simulated oscillation (see Fig. 1a) assumed a periodic concentration increase ${\mathrm{\Delta }}C$ and pulse interval ${t}_P$. However, these parameters might change substantially over time. Hence, an additional simulation was performed with random changes according to a manual ‘noise level’ to both parameters at every interval to test the robustness of the results. This reduced the degree of dominance exhibited by the dominant strain slightly, and the relative abundance of the subordinate strain was increased. This effect scaled with the noise level of random changes but did not change the overall results substantially. A plausible explanation is that random fluctuations of parameters that further favour the already-dominant resistant strain yield only marginal increases because the resistant population is near saturation, whereas even minor positive perturbations for the susceptible strain—starting from a much lower baseline—translate into relatively large gains and thus exert a stronger effect on their population. While this is consistent with general ecology (intermediate disturbance hypothesis) (Connell 1978), it is unclear whether this effect is relevant enough to be observed in practice or is merely theoretical.

**Figure 5.**
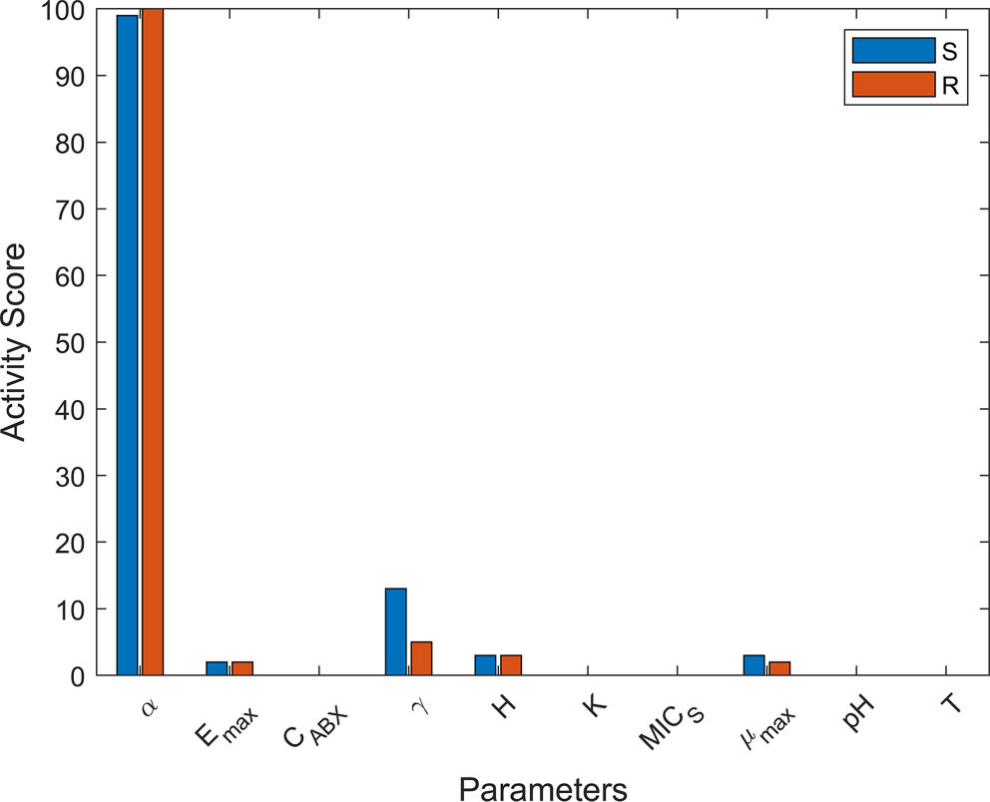
Global activity score of selected parameters towards the mean amount of susceptible bacteria S (blue) and resistant bacteria R (red). Activity scores are expressed as an index between 0 and 100. ${\boldsymbol{\alpha }}$ is the fitness cost, ${{\boldsymbol{E}}}_{{\boldsymbol{max}}}$ is the maximum inhibition rate, ${{\boldsymbol{C}}}_{{\boldsymbol{ABX}}}$ is the antibiotic concentration, γ is the nutrient consumption rate constant, ${\boldsymbol{H}}$ is the Hill coefficient, ${{\boldsymbol{K}}}_{\boldsymbol{m}}{\boldsymbol{\ }}$ is the Monod-coefficient, ${\boldsymbol{MI}}{{\boldsymbol{C}}}_{\boldsymbol{S}}$ is the MIC for the susceptible strain, ${{\boldsymbol{\mu }}}_{{\boldsymbol{max}}}$ is the maximum specific growth rate, ${\boldsymbol{pH}}$ is the pH-value, and ${\boldsymbol{T}}$ is the temperature.

The global sensitivity analysis indicated that only few parameters influence the model results substantially (Fig. 5). The fitness cost $\alpha $ exceeded the impact of all other parameters by far. This is expected, as the fitness cost is essential to the success of both fractions. When the fitness cost is low, resistant bacteria readily outcompete susceptible bacteria, while the same effect is close to unattainable with a very high fitness cost.

Notably, the activity scores of the MIC for susceptible bacteria ($MI{C}_S$) and the antibiotic concentration (${C}_{ABX}$) in the global sensitivity analysis were very low which is unexpected, as $MI{C}_S$ and ${C}_{ABX}$ affect bacterial competition directly and should show similar activity scores to the fitness cost $\alpha $. The local sensitivity analysis (Tables S2 and S3), however, showed a high sensitivity towards both parameters in both scenarios.

Moderate sensitivity was observed for the nutrient-related parameters, the growth efficiency $\gamma $ and the maximum growth rate ${\mu }_{max}$. Although nutrient availability affects overall bacterial growth and influences competitive interactions, it does not alter the MSC. Thus, its impact on the dominance of a particular strain remains secondary to direct competition parameters (e.g. the fitness cost $\alpha $).

In the two-antibiotic model, sensitivity analysis identified the fitness cost α as the sole parameter with a substantial impact. This does not imply that the other parameters did not affect the result; rather, their effects were considerably outweighed by the dominant influence of α. Environmental parameters such as pH and temperature were found to be negligible to the competition of different strains. Within the parameter space examined, variations in these factors did not shift the *R*/*S* ratio. This was also confirmed by the local sensitivity analyses. While more optimal temperatures and pH values facilitated bacterial growth, both strains were equally affected. Thus, future studies should prioritize direct competition and antibiotic-specific parameters to advance the understanding of AMR. Moreover, the apparent insensitivity to pH and temperature suggests that laboratory findings can be transferred to field conditions without major adjustments for these parameters, if the fitness cost is not influenced by pH or temperature. Note that only direct effects of pH and temperature on bacteria were simulated. Effects of these parameters on sorption, dissociation or half-lives were not explored.

For each antibiotic pair, the 2.5th, 50th (median), and 97.5th percentiles were calculated for all calibrated parameters (see Supplementary Data). For the combinations of sulfamethoxazole-trimethoprim and sulfamethoxazole-sulfadiazine, the percentile ranges were very tight, reflecting the high number of replicates. For the combination sulfamethoxazole-chloramphenicol, percentile ranges were much wider, indicating a much lower reliability of the determined parameters. This is likely caused by higher variations in the replicates of this combination. This shows the general robustness of and reliability of the model, as it was able to reflect the low uncertainty resulting from consistent data as well as the high uncertainty resulting from less consistent data.

## Conclusions

The results of our study here show that pulsed antibiotic inputs can substantially increase resistance risks, even when average concentrations remain below the MSC. By integrating antibiotic input dynamics with microbial competition models, we developed a computational framework that provides a transferable tool for quantitative resistance risk assessment, applicable to different release regimes and to other growth-limiting substances (e.g. biocides, heavy metals, etc.). The introduction of the SF further extends the MSC concept to antibiotic combinations, enabling the quantification of potential synergistic effects that might promote resistance even below individual MSCs. Its general applicability, however, depends on validation with additional data, as it has only been tested for two strains from *Acinetobacter baylyi* BD413 and a limited set of antibiotics.

Future work should experimentally validate the predicted effects of antibiotic pulses under environmentally relevant conditions and expand the framework to capture interactions among more than two antibiotics. Extending the new concept of the SF to higher-order mixtures, supported by laboratory studies, will be crucial for a realistic assessment of resistance risks under environmental conditions. These developments will improve the reliability of predictive modelling and provide a basis for mitigation strategies and environmental thresholds.

## Supplementary Material

fiaf128_Supplemental_Files

## Data Availability

The used MATLAB-Code and all data are publicly available at https://www.github.com/Sigma-Librae/PULSE.
