# Supplementary material for: Pulsed antibiotic release into the environment may foster the spread of antimicrobial resistance
Source: FEMS Microbiol Ecol. 2025 Dec 19;102(1):fiaf128. doi: 10.1093/femsec/fiaf128 (PMC12750453; doi:10.1093/femsec/fiaf128)
Supplement: fiaf128_Supplemental_Files [file fiaf128_supplemental_files.zip › Supplementary_data.docx]

**SUPPLEMENTARY DATA**

**Pulsed antibiotic release into the environment may foster the spread of antimicrobial resistance**

Matthias Böckmann^1*^, Katharina Axtmann^2^, Gabriele Bierbaum^2^, Christiane Zarfl^1*^

^1^Eberhard Karls University of Tübingen, Department of Geosciences, Schnarrenbergstraße 94-96, 72076 Tübingen, Germany

^2^ University Hospital Bonn, Institute of Medical Microbiology, Immunology and Parasitology, Venusberg-Campus 1, 53127 Bonn, Germany

***Corresponding authors:** [matthias.boeckmann@uni-tuebingen.de](mailto:matthias.boeckmann@uni-tuebingen.de), [christiane.zarfl@uni-tuebingen.de](mailto:christiane.zarfl@uni-tuebingen.de)

# Full derivation of equations describing antibiotic pulses

At equilibrium, the concentration increase due to a pulse must be equal to the dissipation in between pulses

$$\begin{aligned} \Delta C\overset{eq.}{=}C_{max}-C_{max}\cdot e^{-k\cdot t_{P}}\#\left( S SEQ eq 1 \right) \end{aligned}$$

where $\Delta C$ [ML^-3^] is the concentration increase due to a pulse, $C_{max}$ [ML^-3^] is the maximum concentration directly after the pulse, $k$ is the degradation rate constant [T^-1^] and $t_{P}$ [T] is the time between pulses. The equation is solved for $C_{\max}$:

$$\begin{aligned} \Delta C=C_{\max}\left( 1-e^{-k\cdot t_{P}} \right)\#\left( S SEQ eq 2 \right) \end{aligned}$$

$$\begin{aligned} C_{max}=\frac{\Delta C}{1-e^{-k\cdot t_{P}}}\#\left( S SEQ eq 3 \right) \end{aligned}$$

The dissipation rate constant $k$ is replaced by the expression for the half-life:

$$\begin{aligned} k=\frac{\ln\left( 2 \right)}{t_{\frac{1}{2}}}\#\left( S SEQ eq 4 \right) \end{aligned}$$

$$\begin{aligned} C_{max}=\frac{\Delta C}{1-e^{-\frac{\ln\left( 2 \right)}{t_{\frac{1}{2}}}\cdot t_{P}}}\#\left( S SEQ eq 5 \right) \end{aligned}$$

where $t_{\frac{1}{2}}$ [T] is the substance-specific half-life.

The ratio between $t_{P}$ and $t_{\frac{1}{2}}$ is then defined as the dimensionless factor $n$ [-]:

$$\begin{aligned} \frac{t_{P}}{t_{\frac{1}{2}}}=n\#\left( S SEQ eq 6 \right) \end{aligned}$$

$$\begin{aligned} C_{max}=\frac{\Delta C}{1-e^{-\ln\left( 2 \right)\cdot n}}\#\left( S SEQ eq 7 \right) \end{aligned}$$

$$\begin{aligned} C_{max}=\frac{\Delta C}{1-\left( e^{\ln\left( 2 \right)} \right)^{-n}}\#\left( S SEQ eq 8 \right) \end{aligned}$$

$$\begin{aligned} C_{max}=\frac{\Delta C}{1-2^{-n}}=\Delta C\cdot\frac{1}{1-2^{-n}}\#\left( S SEQ eq 9 \right) \end{aligned}$$

$C_{\min}$ [ML^-3^] is the minimum concentration once the oscillation reaches equilibrium:

$$\begin{aligned} C_{\min}=C_{max}-\Delta C\#\left( S SEQ eq 10 \right) \end{aligned}$$

$$\begin{aligned} C_{\min}= \Delta C\cdot\left( \frac{1}{1-2^{-n}}-1 \right)\#\left( S SEQ eq 11 \right) \end{aligned}$$

The average of any function $f$ over the interval from $a$ to $b$ is defined as:

$$\begin{aligned} \frac{\int_{a}^{b} f\left( x \right) dx}{b-a}\#\left( S SEQ eq 12 \right) \end{aligned}$$

Hence, the average value $\bar{C}$ [ML^-3^] between pulses is defined as:

$$\begin{aligned} \bar{C}=\frac{\int_{0}^{t_{P}} C_{max}\cdot e^{-k\cdot t} dt}{t_{P}-0}\#\left( S SEQ eq 13 \right) \end{aligned}$$

where the time of the pulse is $t=0$. The integral of the first-order degradation is defined as:

$$\begin{aligned} \int C_{max}\cdot e^{-k\cdot t} dt=-C_{max}\cdot\frac{1}{k}\cdot e^{-k\cdot t}\#\left( S SEQ eq 14 \right) \end{aligned}$$

Inserted into the expression for $\bar{C}$:

$$\begin{aligned} \bar{C}=\frac{-C_{max}\cdot\frac{1}{k}\cdot e^{-k\cdot t_{P}}-\left( -C_{max}\cdot\frac{1}{k} \right)}{t_{P}}\#\left( S SEQ eq 15 \right) \end{aligned}$$

$$\begin{aligned} \bar{C}=\frac{C_{max}\cdot\frac{1}{k}\cdot\left( 1-e^{-k\cdot t_{P}} \right)}{t_{P}}\#\left( S SEQ eq 16 \right) \end{aligned}$$

The dissipation rate constant $k$ is replaced by the expression for the half-life:

$$\begin{aligned} k=\frac{\ln\left( 2 \right)}{t_{\frac{1}{2}}}\#\left( S SEQ eq 17 \right) \end{aligned}$$

$$\begin{aligned} \bar{C}=\frac{C_{max}\cdot\left( 1-e^{-\frac{\ln\left( 2 \right)}{t_{\frac{1}{2}}}\cdot t} \right)}{t_{P}}\cdot\frac{t_{\frac{1}{2}}}{\ln\left( 2 \right)}\#\left( S SEQ eq 18 \right) \end{aligned}$$

The ratio between $t_{P}$ and $t_{\frac{1}{2}}$ is then defined as the dimensionless factor $n$ [-]:

$$\begin{aligned} \frac{t_{P}}{t_{\frac{1}{2}}}=n\#\left( S SEQ eq 19 \right) \end{aligned}$$

$$\begin{aligned} \bar{C}=\frac{C_{max}\cdot\left( 1-e^{-\ln\left( 2 \right)\cdot n} \right)}{n\cdot\ln\left( 2 \right)}\#\left( S SEQ eq 20 \right) \end{aligned}$$

$$\begin{aligned} \bar{C}=\frac{C_{max}\cdot\left( 1-2^{-n} \right)}{n\cdot\ln\left( 2 \right)}\#\left( S SEQ eq 21 \right) \end{aligned}$$

$C_{max}$ is replaced by the previously derived expression:

$$\begin{aligned} C_{max}=\Delta C\cdot\frac{1}{1-2^{-n}}\#\left( S SEQ eq 22 \right) \end{aligned}$$

$$\begin{aligned} \bar{C}=\frac{\Delta C\cdot\frac{1}{1-2^{-n}}\cdot\left( 1-2^{-n} \right)}{n\cdot\ln\left( 2 \right)}\#\left( S SEQ eq 23 \right) \end{aligned}$$

$$\begin{aligned} \bar{C}=\frac{\Delta C}{n\cdot\ln\left( 2 \right)}\#\left( S SEQ eq 24 \right) \end{aligned}$$

$$\begin{aligned} \bar{C}=\Delta C\cdot\frac{1}{\ln\left( 2 \right)\cdot n}\#\left( S SEQ eq 25 \right) \end{aligned}$$

# Sensitivity and uncertainty analysis

Table S1 Simulations of bacterial competition under pulsed antibiotic exposure. For each simulation, the mean resistant population is divided by the susceptible population (R/S ratio). Each shown value is the average of 2000 simulations. Pulse interval $t_{P}$ and pulse increase $\Delta C$ were randomly perturbed by adding or subtracting a random value, drawn from a uniform probability distribution ranging from 0 to the product of the noise level and the respective parameter value.

| **Noise level** | **R/S ratio (susceptible strain dominates)** | **R/S ratio (resistant strain dominates)** |
| --- | --- | --- |
| 0 | 0.47 | 3.05 |
| 0.1 | 0.48 | 3.03 |
| 0.2 | 0.49 | 3.00 |
| 0.3 | 0.53 | 2.92 |
| 0.4 | 0.56 | 2.84 |
| 0.5 | 0.60 | 2.77 |

Table S2 Results of local sensitivity analysis for the case with a single antibiotic. Parameters were varied by 10% of the chosen value

| **Parameter** | $\boldsymbol{\Delta}$**S [%]** | | $\boldsymbol{\Delta}\boldsymbol{R}$ **[%]** | |
| --- | --- | --- | --- | --- |
|  | **+10%** | **-10%** | **+10%** | **-10%** |
| $\alpha$ | 5.1 | -8.1 | -24.8 | 39.6 |
| $E_{max}$ | -4.9 | 3.4 | 23.8 | -16.9 |
| $C_{antibiotic}$ | -9.7 | 5.2 | 47.7 | -25.6 |
| $\gamma$ | -9.1 | 11.2 | -8.4 | 10.2 |
| $H$ | 4.7 | -9.2 | -23 | 45 |
| $K$ | 0.0 | 0.0 | 0.1 | 0.0 |
| $MIC_{S}$ | 4.9 | -11.3 | -23.9 | 55.2 |
| $\mu_{max}$ | 0.0 | 0.0 | 0.0 | 0.1 |
| $T$ | 0.0 | 0.0 | 0.0 | 0.0 |
| $pH$ | 0.0 | 0.0 | 0.0 | 0.0 |

Table S3 Percentile ranges (2.5^th^, 50^th^, and 97.5^th^ percentiles) of calibrated model parameters for antibiotic combinations based on bootstrap analysis.

| **Parameter** | **Trimethoprim-Sulfamethoxazole** | | | **Sulfadiazine-Sulfamethoxazole** | | | **Chloramphenicol- Sulfamethoxazole** | | |
| --- | --- | --- | --- | --- | --- | --- | --- | --- | --- |
|  | **2.5^th^ percentile** | **Median** | **97.5^th^ percentile** | **2.5^th^ percentile** | **Median** | **97.5^th^ percentile** | **2.5^th^ percentile** | **Median** | **97.5^th^ percentile** |
| $\alpha$ | 0.50 | 0.50 | 0.50 | 0.46 | 0.46 | 0.47 | 0.46 | 0.47 | 0.50 |
| $\lambda$ | 3.99 | 4.20 | 4.30 | -1.59 | -1.26 | -0.93 | -3.34 | 5.79 | 8.47 |
| $E_{S,1}$ | 11.20 | 11.26 | 12.03 | 19.66 | 19.83 | 20.00 | 11.40 | 15.20 | 31.54 |
| $E_{S,2}$ | 0.15 | 0.15 | 0.16 | 0.06 | 0.10 | 0.15 | 0.01 | 0.02 | 0.04 |
| $E_{R,1}$ | 0.00 | 0.04 | 0.48 | 0.00 | 0.07 | 0.13 | 9.35 | 24.76 | 38.24 |
| $E_{R,2}$ | 38.22 | 38.23 | 38.24 | 5.17 | 21.71 | 38.24 | 1.12 | 8.59 | 18.57 |

# Individual datasets

The following plots present the individual R/S ratio datapoints obtained from the checkerboard wells, with each plot corresponding to a single checkerboard. Antibiotic combinations are abbreviated in the titles as follows: SMX = sulfamethoxazole, TRI = trimethoprim, SDZ = sulfadiazine, and CMP = chloramphenicol. Due to data normalization, the highest and lowest values are reported as NaN (not-a-number), while additional NaN entries denote defective wells.

## Trimethoprim-Sulfamethoxazole

| 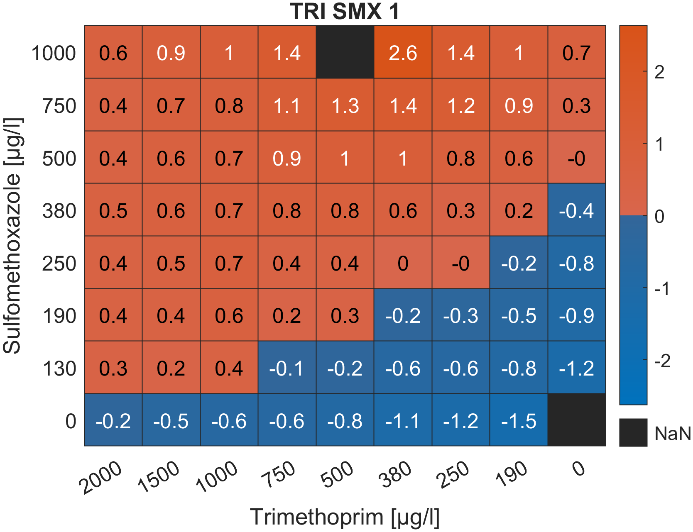 | 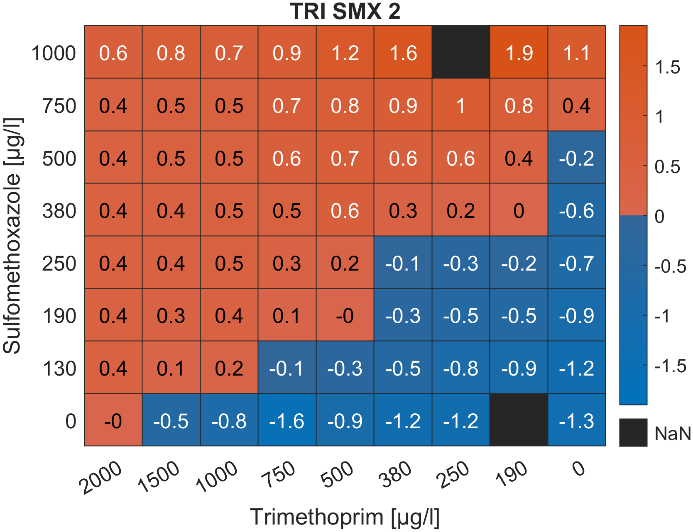 |
| --- | --- |
| 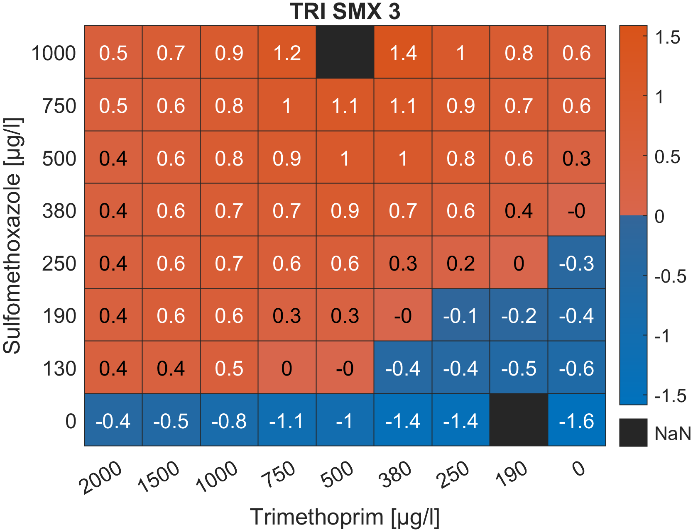 | 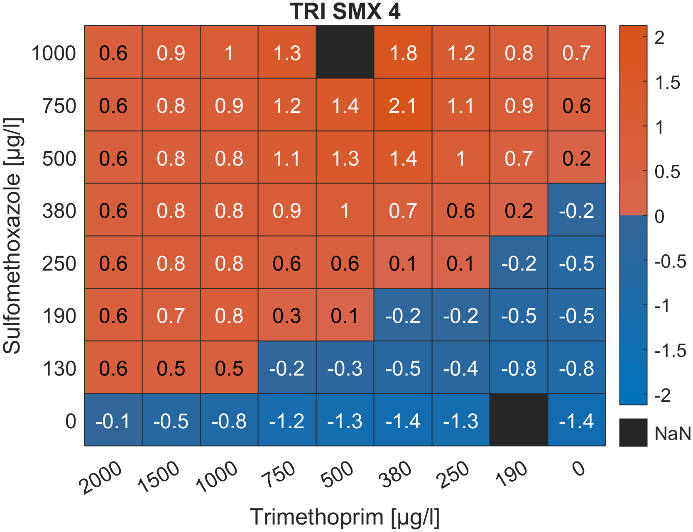 |
| 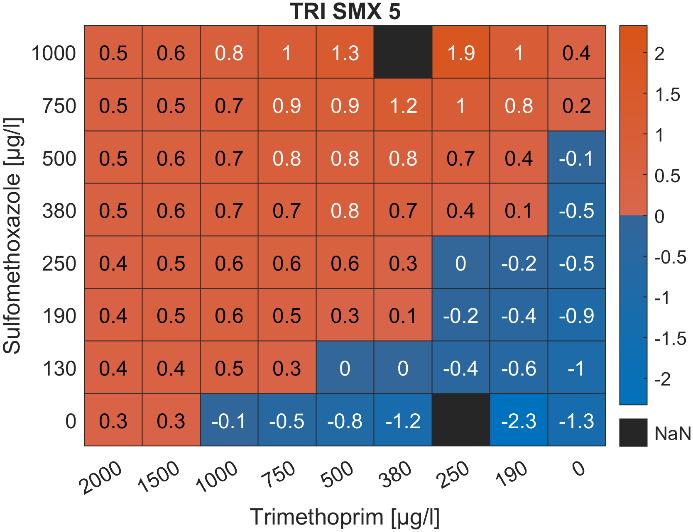 | 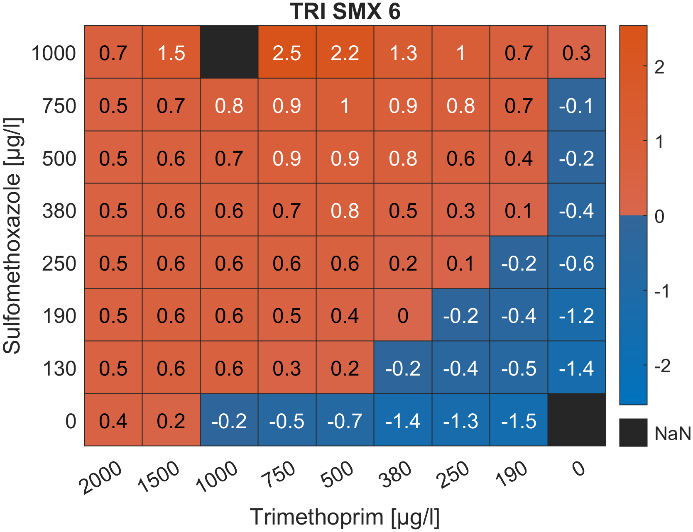 |
| 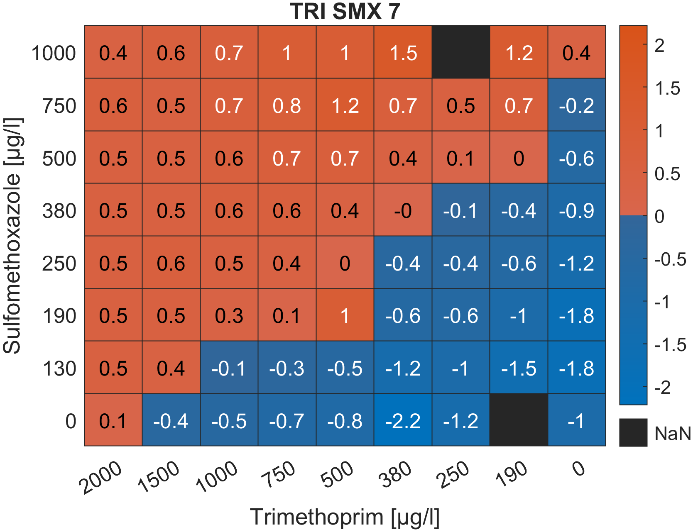 | 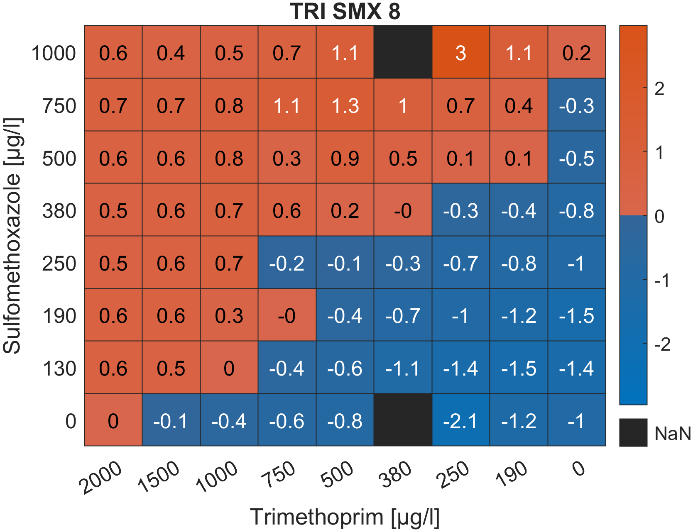 |
| 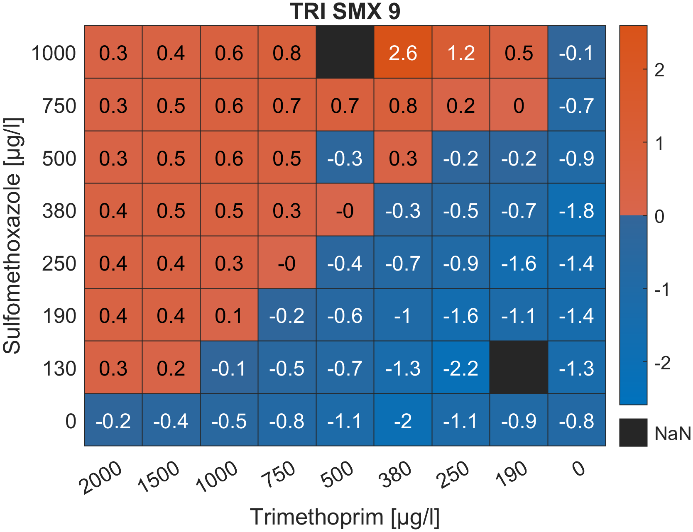 | 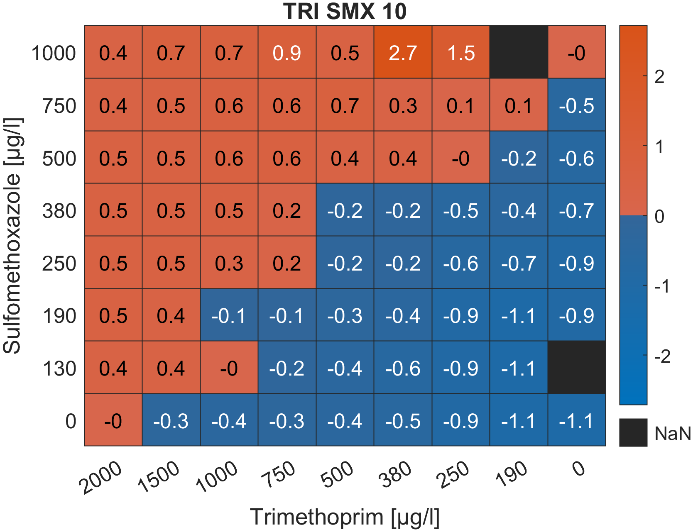 |

## Sulfadiazine-Sulfamethoxazole

| 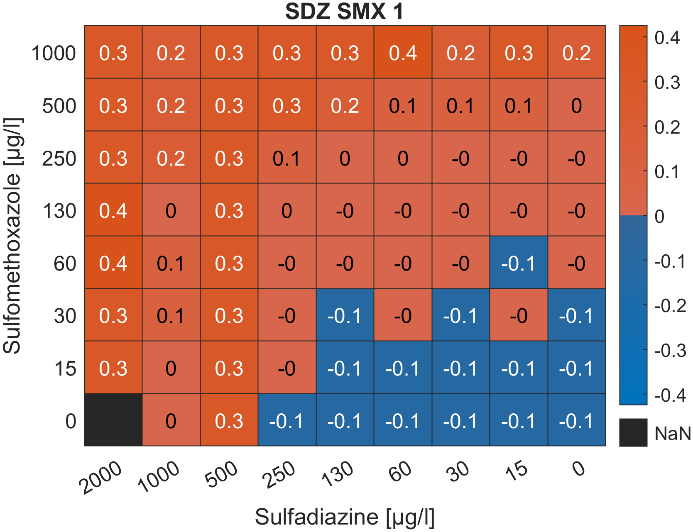 | 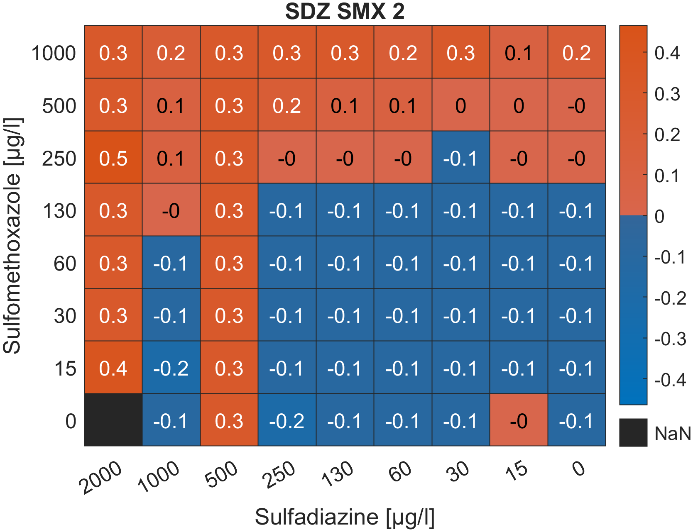 |
| --- | --- |
| 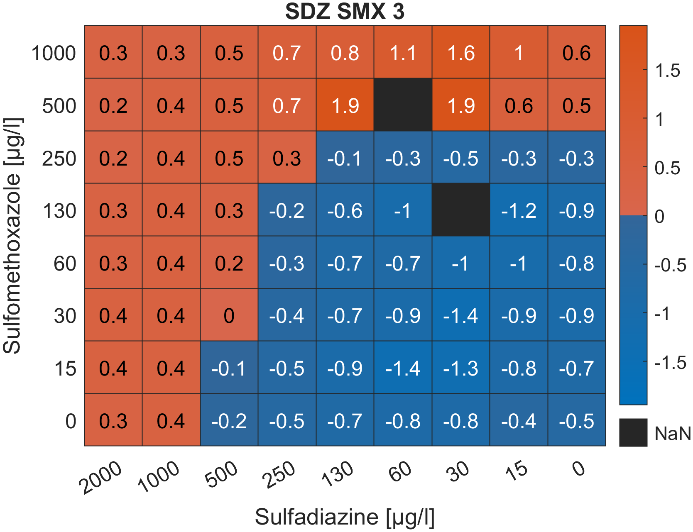 | 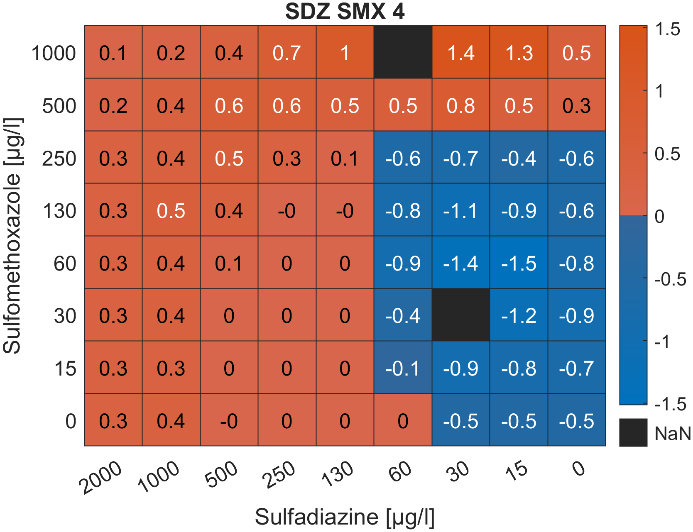 |
| 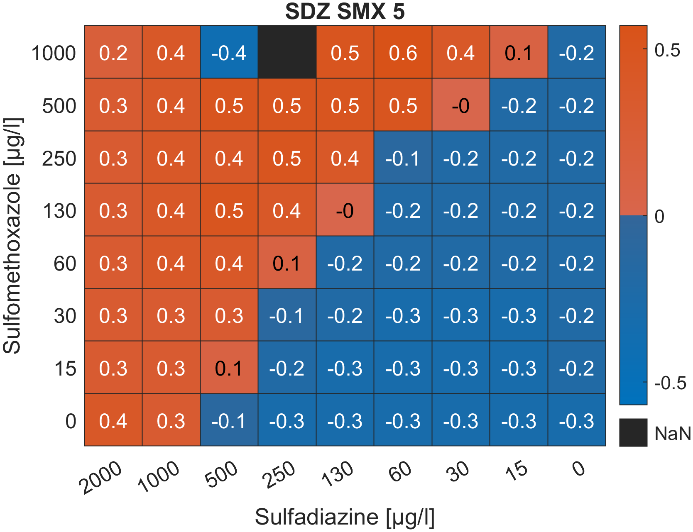 | 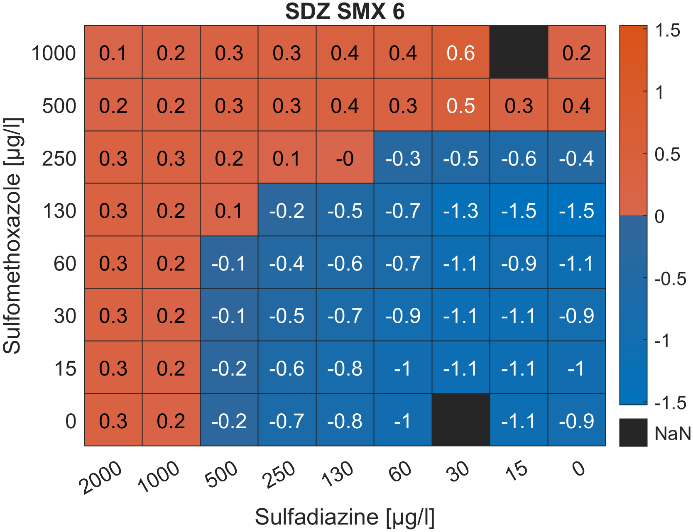 |
| 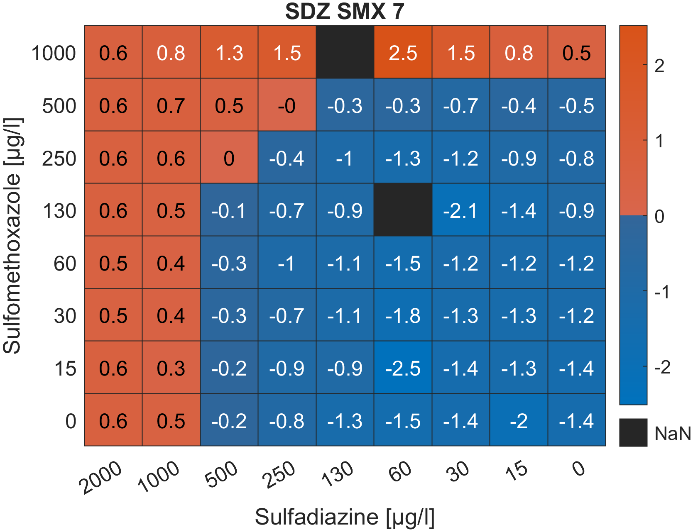 | 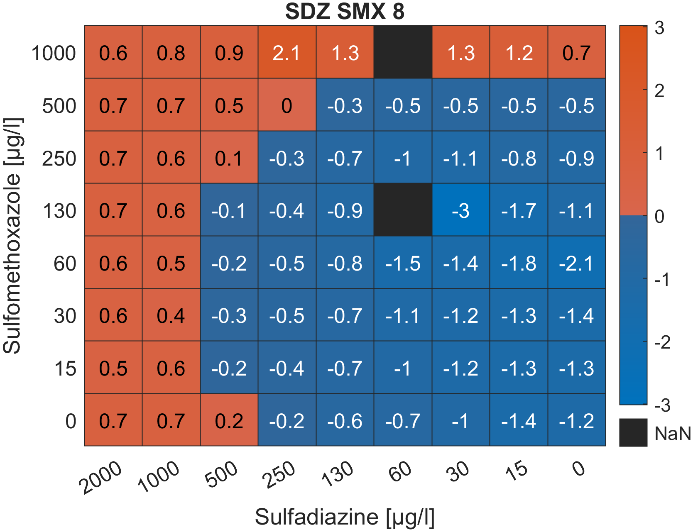 |
| 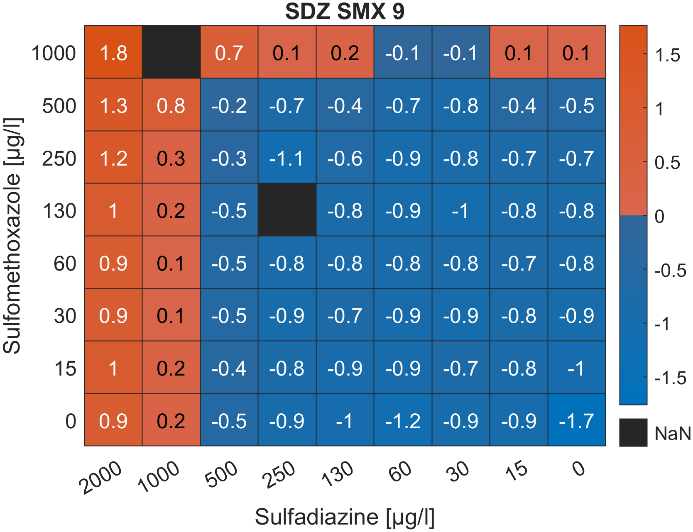 | 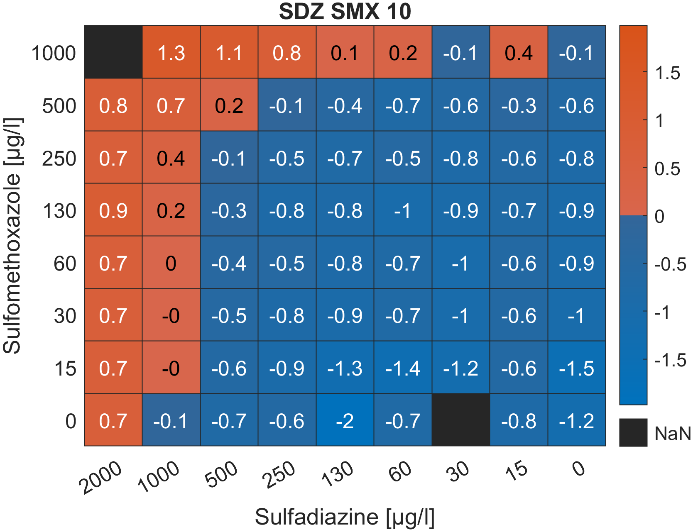 |
| 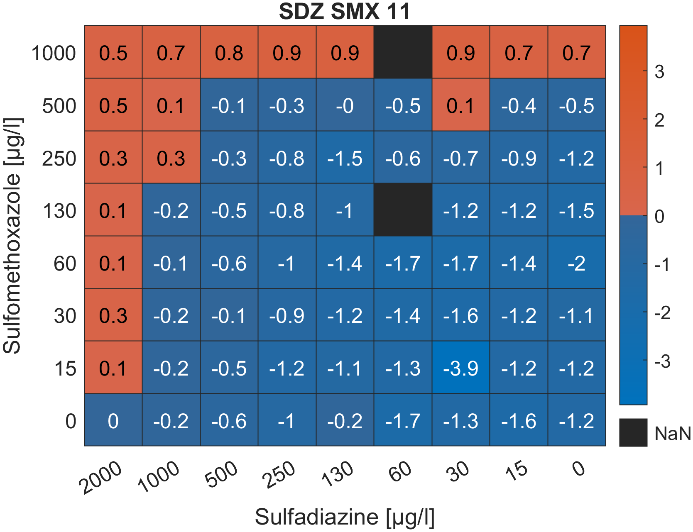 | 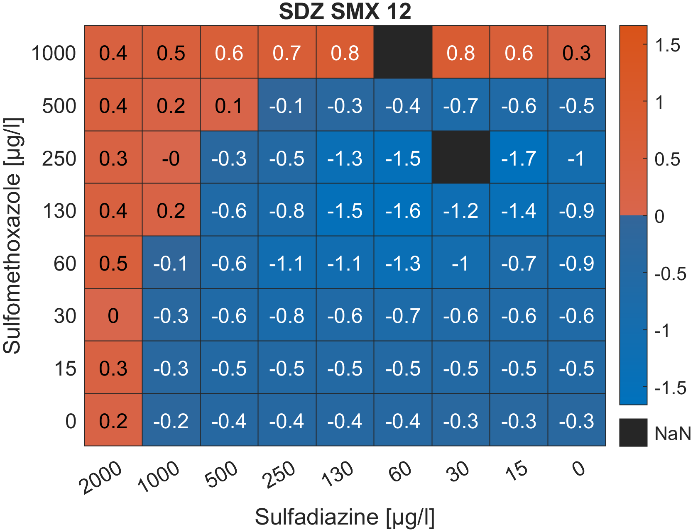 |
| 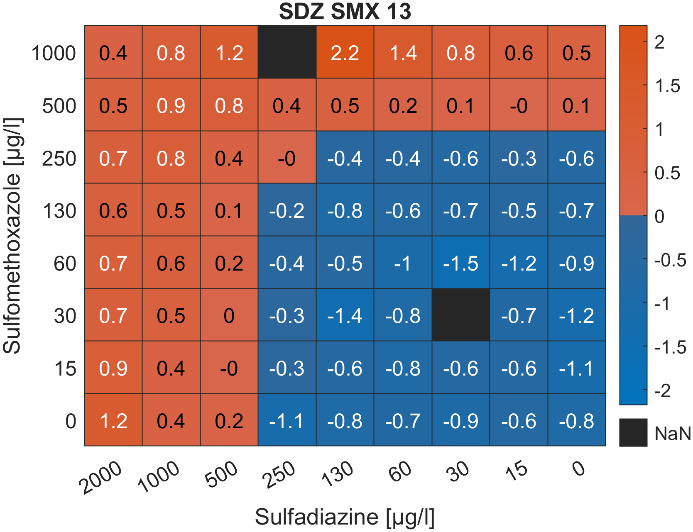 | 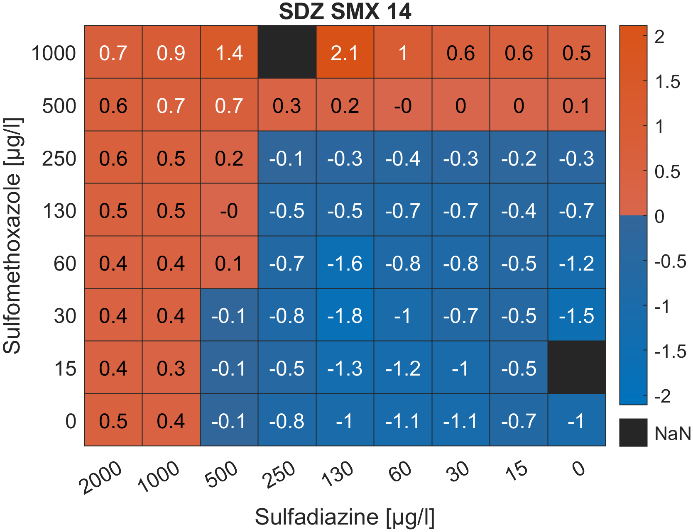 |
| 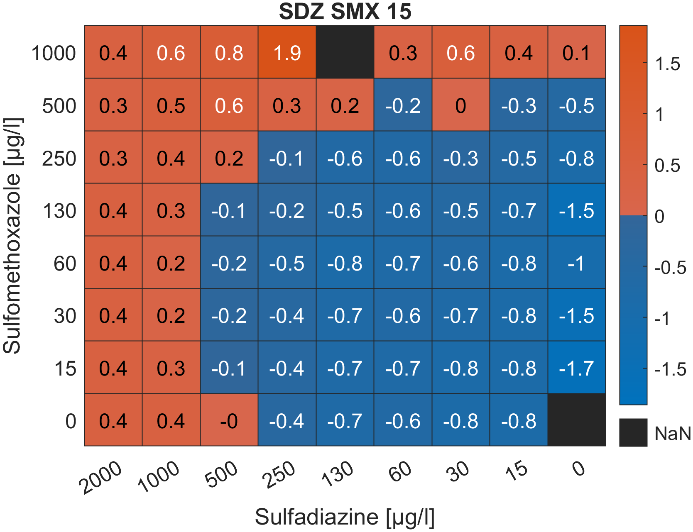 | 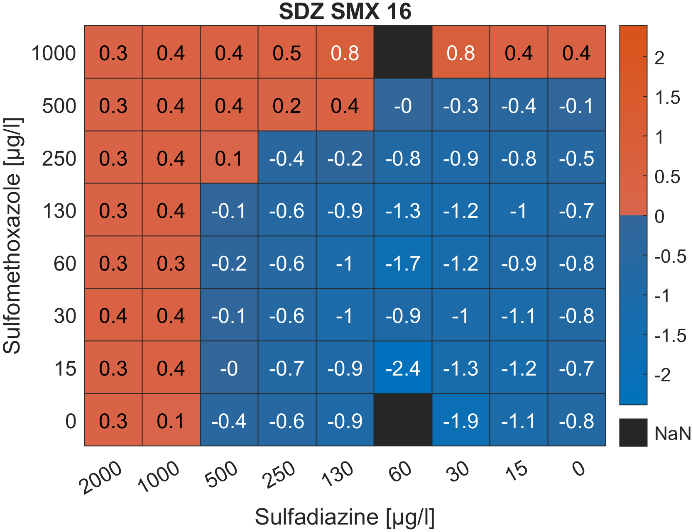 |

## Chloramphenicol-Sulfamethoxazole

| 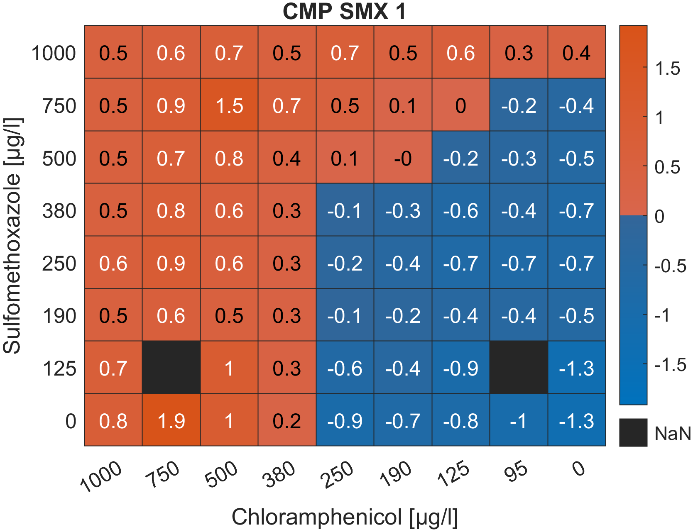 | 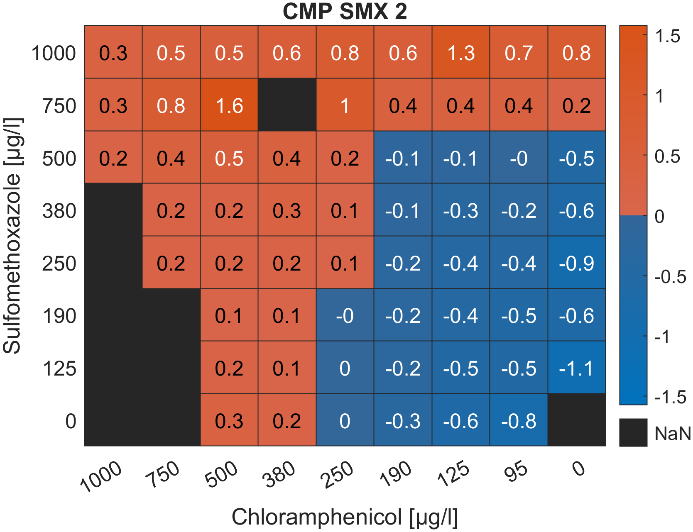 |
| --- | --- |
| 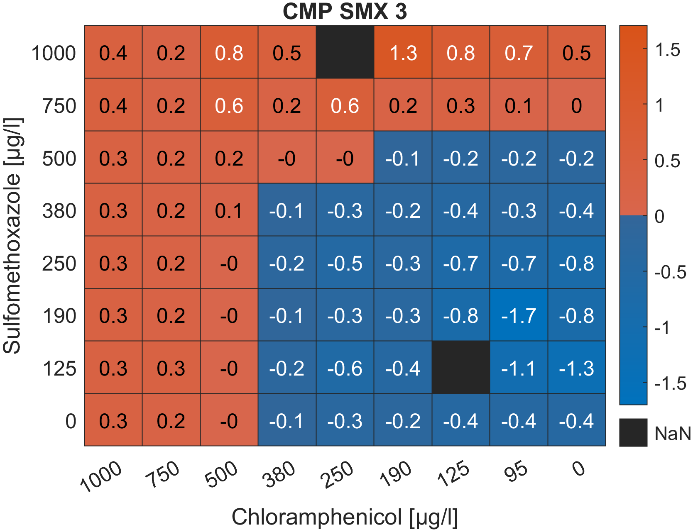 | 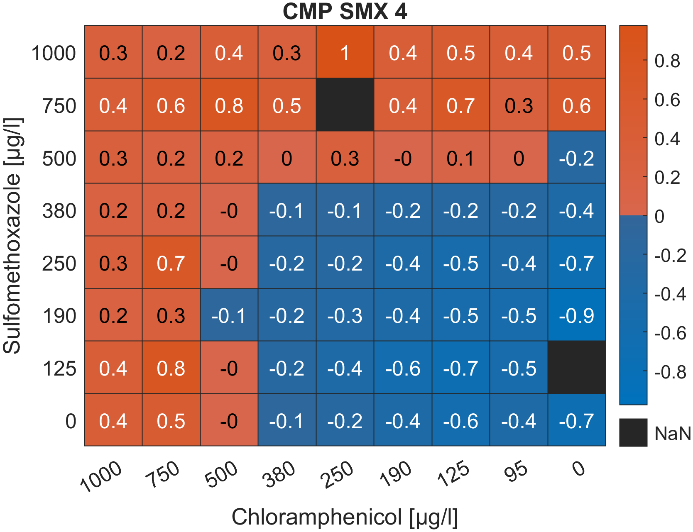 |
| 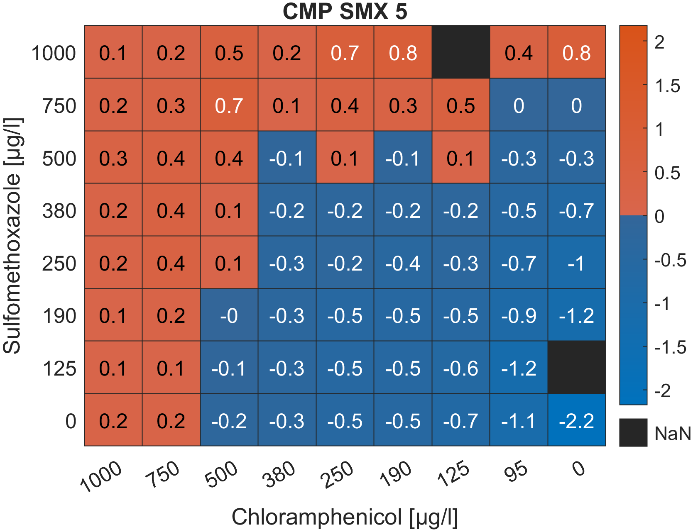 | 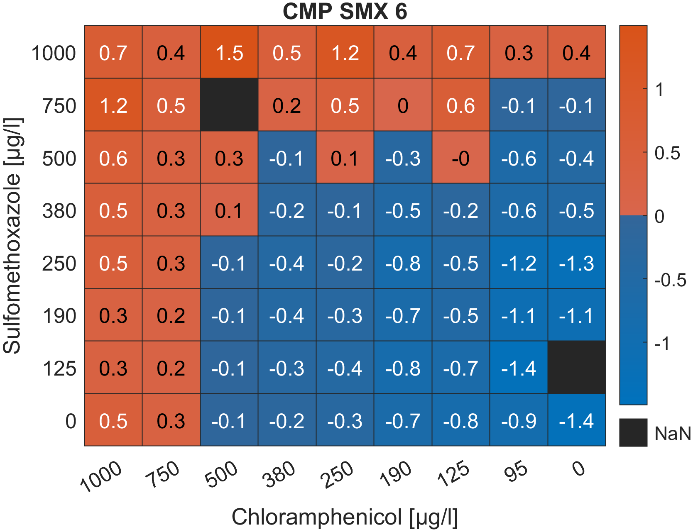 |
| 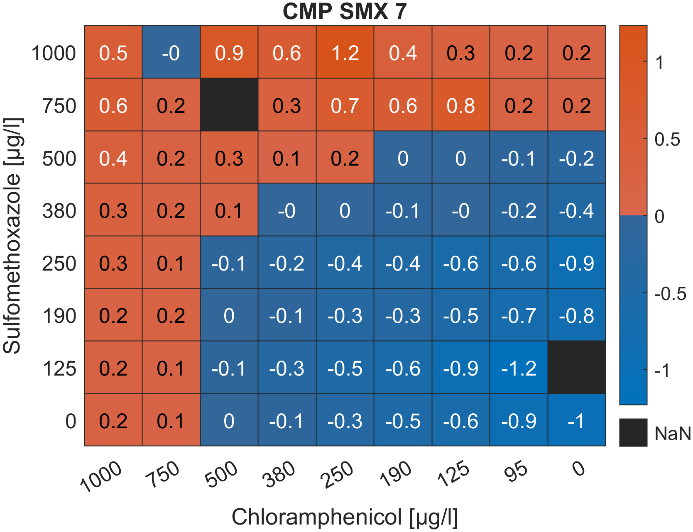 | 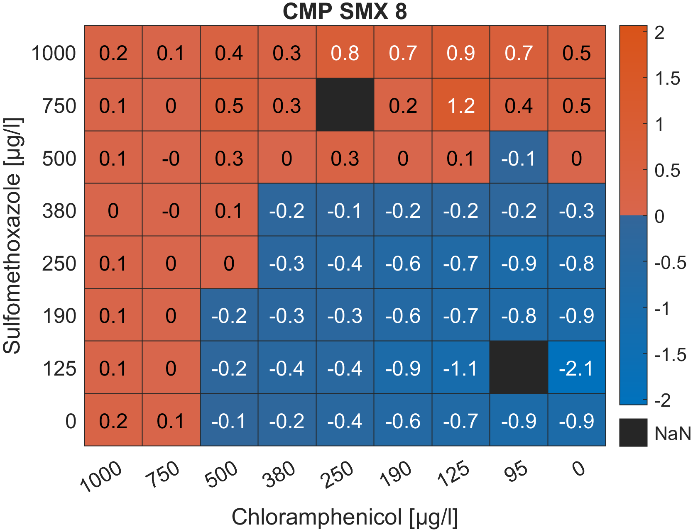 |
| 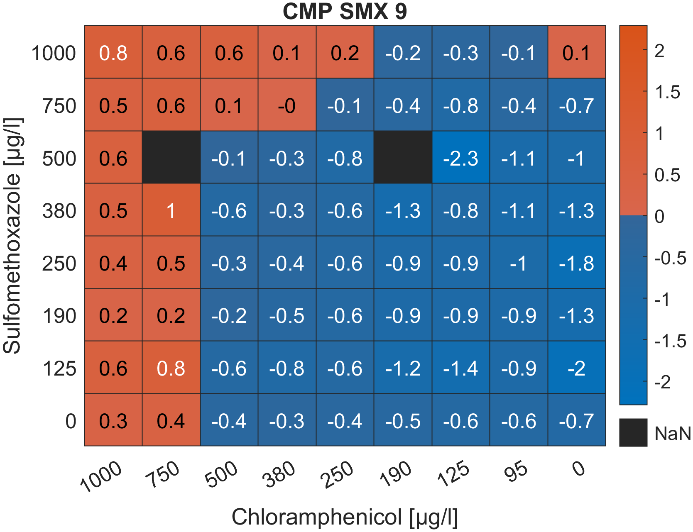 | 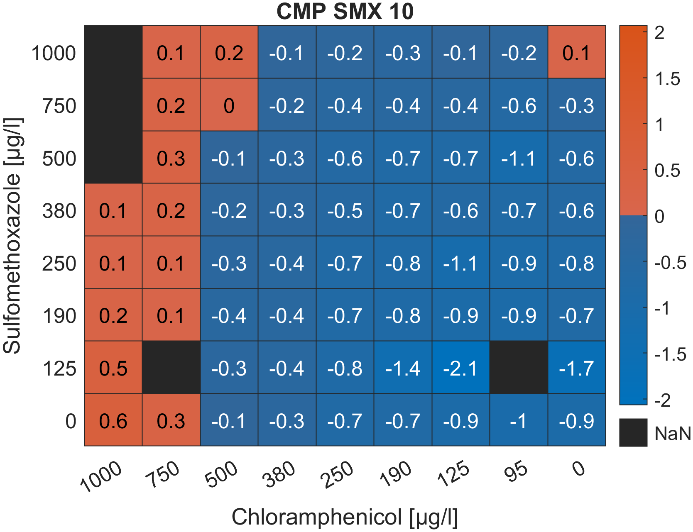 |
